# Disability as a neglected outcome of neglected tropical diseases: A systematic review

**DOI:** 10.1371/journal.pntd.0014480

**Published:** 2026-07-01

**Authors:** Caio Cesar Leiva Bastos Barrionuevo, Jefferson da Silva Valente, Bernardo Maia da Silva, Cássia da Luz Goulart, Alex Maciel, Aldair Darlan Santos-de-Araújo, Camila Miriam Suemi Sato Barros do Amaral, Eduardo Fernandes da Silva Junior, Stephanie Vitória Alves dos Santos, Erika Gomes, Nadia Cubas-Vega, Guilherme Peixoto Tinoco Arêas, Fernando Almeida-Val

**Affiliations:** 1 Universidade do Estado do Amazonas, Manaus, Amazonas, Brazil; 2 Fundação de Medicina Tropical Dr Heitor Vieira Dourado, Manaus, Amazonas, Brazil; 3 Hospital Universitário Getúlio Vargas, Programa de Residência em Fisioterapia Hospitalar, Manaus, Amazonas, Brazil; 4 Universidade Federal de São Carlos, São Carlos, São Paulo, Brazil; 5 Fundação de Hematologia e Hemoterapia do Amazonas, Manaus, Amazonas, Brazil; 6 Fundación Cristianos Asistiendo a Quemados, Tegucigalpa, Honduras; 7 Universidade Federal do Amazonas, Manaus, Amazonas, Brazil; 8 Universidade Nilton Lins (UNL), Manaus, Amazonas, Brazil; 9 Departamento de Saúde Coletiva da Faculdade de Medicina, Universidade do Estado do Amazonas, Manaus, Amazonas, Brazil; Beijing Children's Hospital Capital Medical University, CHINA

## Abstract

**Background:**

Neglected tropical diseases (NTDs) affect more than one billion people worldwide, predominantly in low-resource settings. While substantial progress has been made in reducing transmission and mortality, the long-term disabilities resulting from NTDs remain insufficiently recognized and inadequately addressed within control programs.

**Objective:**

To identify, describe, and systematize the types of disabilities reported among individuals affected by NTDs, providing a comprehensive overview of the magnitude and diversity of long-term functional impairments associated with these conditions.

**Methods:**

This systematic review was conducted in accordance with PRISMA 2020 guidelines. A comprehensive search was performed in PubMed, Cochrane Central, Embase, CINAHL, LILACS, and Web of Science databases, without restrictions on language or publication date. Studies reporting long-term disabilities or sequelae in individuals diagnosed with WHO-listed NTDs were included. Data on study characteristics, population demographics, and disability types were extracted and synthesized descriptively. Methodological quality was assessed using design-specific tools (MINORS, CARE checklist, and Jadad scale).

**Results:**

The initial search identified 958 records across databases. After removal of duplicates (n = 478), 480 records were screened, of which 310 were assessed for full-text eligibility. Following the application of inclusion and exclusion criteria, 130 studies were included in the final analysis, encompassing 551,574 individuals from 25 countries. Overall, 29% (n = 158,104) of evaluated individuals presented with at least one form of disability. Physical and motor impairments were the most prevalent (24.6%, n = 135,683), predominantly associated with leprosy-related neuropathic and musculoskeletal sequelae. Visual impairment was the second most frequent disability (1.81%, n = 10,007), largely attributable to trachoma and onchocerciasis. Cardiovascular, neurological, psychological, and social impairments were also reported. Considerable heterogeneity and underreporting of disability outcomes were observed across studies.

**Conclusions:**

Disability represents a substantial and underappreciated outcome of NTDs, extending far beyond infection-related morbidity. The high burden of long-term physical, visual, and other functional impairments underscores the urgent need to integrate disability assessment, rehabilitation services, and disability-inclusive development into NTD control strategies. Addressing these gaps is essential to advance patient-centered care and achieve the broader goals of the WHO NTD Roadmap and Universal Health Coverage.

## Author summary

Neglected tropical diseases (NTDs) affect more than one billion people worldwide, primarily in low-resource settings. While global control efforts have focused on reducing transmission and mortality, the long-term disabilities caused by these diseases remain poorly described and rarely incorporated into public health strategies. In this systematic review, we analyzed 130 studies including 551,574 individuals across 25 countries to identify the types and frequency of disabilities associated with NTDs. Overall, 29% of individuals experienced at least one disability. Physical and motor impairments were the most common, largely related to leprosy, followed by visual impairment associated with trachoma and onchocerciasis. Cardiovascular, neurological, and psychosocial consequences were also reported. These findings highlight that disability is a substantial and underrecognized outcome of NTDs, extending beyond infection control. The evidence also revealed considerable heterogeneity in how disability is defined and measured, as well as underreporting across diseases. Our results emphasize the need to integrate disability assessment, rehabilitation services, and disability-inclusive policies into NTD programs. Addressing long-term functional outcomes is essential to improve quality of life and to achieve the goals of Universal Health Coverage and the WHO NTD Roadmap.

## Introduction

It is estimated that more than one billion people are affected by neglected tropical diseases (NTDs), which are prevalent in tropical and subtropical regions and are often associated with poverty and poor sanitation infrastructure [1,2]. Each year, approximately 1.5 billion people require preventive or therapeutic interventions, underscoring the persistence of these diseases as a major global public health challenge and the need for coordinated action among governments, international organizations, and communities [1–4]. In response, the World Health Organization (WHO) developed the NTDs 2021–2030 roadmap, proposing a shift from isolated vertical programs toward integrated and transversal strategies aimed at reducing by 90% the number of people requiring treatment, eradicating diseases, and eliminating at least one NTD in 100 countries by 2030 [5]. These strategies emphasize coordinated interventions such as vector control and improvements in water, sanitation, and hygiene [5].

NTDs are characterized by marked clinical heterogeneity, with manifestations ranging from acute to chronic and from localized to systemic conditions [2,6]. Their frequently silent or insidious progression, combined with diverse signs and symptoms, hampers early diagnosis and increases morbidity, particularly among vulnerable populations with limited access to healthcare services [7–10]. Beyond their immediate clinical effects, NTDs are strongly associated with chronic disabilities and social stigmatization, leading to discrimination, social exclusion, and substantial economic and social consequences [7,11–16].

Physical and functional disabilities represent some of the most significant long-term consequences of NTDs. These impairments can compromise activities of daily living, reduce mobility and work capacity, and result in persistent disabling symptoms that undermine individual autonomy [2,6]. Often underestimated, such disabilities not only perpetuate cycles of poverty and exclusion but also impose a considerable burden on health systems, particularly in low-resource settings where access to rehabilitation services is limited [2,6]. Delayed diagnosis and inadequate treatment further exacerbate functional decline, increasing the need for long-term care. In this context, effective NTD control strategies, such as elimination programs, mass drug administration, and educational initiatives, depend on a robust understanding of regional and local dynamics, identification of at-risk populations, continuous monitoring of health indicators, and the availability of high-quality scientific evidence to inform policy and practice.

Despite this burden, disability assessment planning and structured rehabilitation strategies remain largely absent from NTD control programs. The persistence of physical and functional disabilities continues to limit affected individuals’ access to education, employment, income generation, and community participation. This challenge is compounded by scarce and fragmented data, underreporting of disabilities, and substantial methodological heterogeneity across studies, which hinder the consolidation of evidence. Therefore, this systematic review hypothesizes that disabilities associated with NTDs have been described in a fragmented and poorly standardized manner in the literature, limiting the understanding of their magnitude and complexity. The objective of this review is to identify and describe the types of disabilities reported among individuals affected by NTDs in peer-reviewed published literature, contributing to a clearer and more comprehensive understanding of the scope of this problem worldwide.

## Methods

### Study design and reporting standards

This systematic review was conducted and reported in accordance with the Preferred Reporting Items for Systematic Reviews and Meta-Analyses (PRISMA 2020) guidelines [17]. The objective was to identify and synthesize evidence on long-term disabilities and sequelae associated with NTDs, regardless of study design, geographic setting, language, or year of publication. The review protocol was registered in the International Prospective Register of Systematic Reviews (PROSPERO under the registration number CRD420261351709).

### Research question

The research question was structured using a PICO-adapted framework appropriate for descriptive systematic reviews, in which the population comprised individuals diagnosed with WHO-listed neglected tropical diseases; the exposure corresponded to the presence of long-term disability or sequelae; no comparison group was defined due to the descriptive nature of the synthesis; and the outcomes included the type and prevalence of disability.

### Search strategy and data sources

A comprehensive literature search was performed in May 2025 across the following electronic databases: PubMed (MEDLINE), Cochrane Central Register of Controlled Trials (CENTRAL), Embase (Elsevier), CINAHL, Latin American and Caribbean Health Sciences Literature (LILACS), and Web of Science (Clarivate Analytics). The search strategy combined Medical Subject Headings (MeSH) and entry terms related to NTDs as defined by the WHO [1], together with terms related to disability, sequelae, functional impairment, and loss of function, using Boolean operators (AND, OR, NOT), in english, spanish and portuguese. No restrictions were applied regarding language or publication date. To ensure completeness, backward and forward citation searches were conducted by screening the reference lists of included studies and relevant systematic reviews. The full search strategy, including all MeSH terms and entry terms for WHO-listed NTDs, is provided in the Supplementary Material.

Title and abstract screening and full-text eligibility assessment were independently performed by two reviewers. Disagreements were resolved by discussion and, when necessary, adjudication by a third reviewer. Prior to screening, a calibration exercise was conducted using a random sample of studies to ensure consistency in the application of eligibility criteria. Agreement metrics (e.g., Cohen’s kappa) were not prospectively calculated. However, duplicate screening, calibration training, and consensus procedures were implemented to minimize selection errors, consistent with PRISMA 2020 recommendations.

### Eligibility criteria

We included observational studies (cross-sectional, case-control, and cohort studies), experimental studies (including clinical trials), ecological studies, and case reports or case series that evaluated individuals of any age diagnosed with one or more WHO-listed NTDs and reported long-term sequelae or disabilities attributable to these conditions. Studies from all geographic regions were eligible for inclusion. The primary outcome of interest was the presence of permanent or long-term disability detected or reported after NTD diagnosis, whether directly resulting from the disease or from its secondary consequences. Studies were excluded if they did not include individuals affected by NTDs, did not report any form of disability or sequelae, involved non-human subjects, or were limited to reviews, editorials, letters, conference abstracts, posters, or grey literature. Articles for which full texts could not be obtained after attempts to contact the authors were also excluded.

### Definition of disability and sequelae

For the purposes of this review, sequelae were defined as permanent anatomical or functional impairments resulting from NTDs. These included physical and motor disabilities; neurological impairments (including sensory, visual, auditory, or cognitive deficits); deformities of limbs or body segments; and chronic damage to organs or body systems that resulted in long-term functional limitations. This definition was intentionally broad to accommodate the heterogeneity of reporting across studies and to capture the multidimensional nature of disability associated with NTDs.

### Study selection

**Fig 1:**
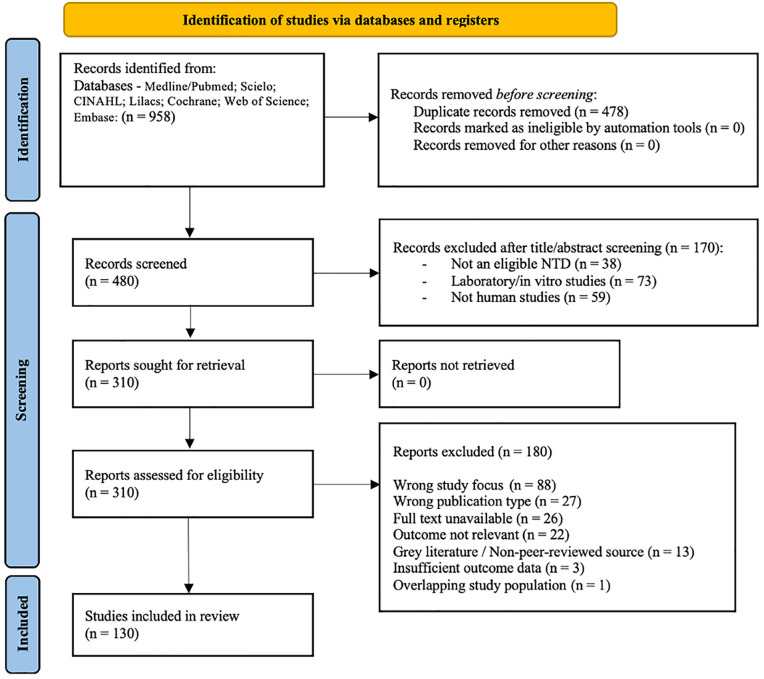
PRISMA flowchart of study selection. Flow diagram showing the number of records identified, screened, excluded, and finally included in the systematic review.

The initial search strategy identified 958 records. After removing duplicates (n = 478), 480 records were screened independently by two reviewers (ADS and JSV) based on titles and abstracts. Disagreements were resolved by consensus, and when necessary, a third reviewer (CCLBB) was consulted. During the screening phase, 170 records were excluded. A total of 310 full-text articles were then assessed for eligibility by ADS and JSV. Of these, 180 were excluded for reasons including non-eligible publication types (reviews, editorials, letters, short reports, and comments), laboratory or in vitro studies, absence of NTDs, or lack of reported sequelae. Ultimately, 130 studies were included in the final analysis (Fig 1).

### Data extraction

Data extraction was independently performed by two reviewers using a standardized and piloted form. Extracted variables included study characteristics (author, year, country, income level, and design), population characteristics (sample size, age, sex, comorbidities, and co-infections), type of neglected tropical disease, disability outcomes, and number of individuals affected. Discrepancies were resolved by discussion and, when necessary, adjudication by a third reviewer. Duplicate extraction was implemented to minimize errors and improve data accuracy.

### Risk of bias and methodological quality assessment

Given the heterogeneity of study designs, methodological quality was assessed using design-specific tools. Observational studies were evaluated using the Methodological Index for Non-Randomized Studies (MINORS) [18,19], while case reports and case series were assessed using the CARE checklist [19]. Randomized clinical trials were evaluated using the Jadad scale [20]. Rather than excluding studies based on quality scores, all eligible studies were retained to provide a comprehensive overview of the evidence. Methodological limitations were considered during interpretation of results. A summary of the risk of bias assessment is provided in the Supplementary Material.

### Data synthesis

Due to substantial heterogeneity in study designs, populations, disability definitions, and outcome measures, a meta-analysis was not performed. Results were synthesized descriptively, focusing on the prevalence and types of disabilities reported across NTDs, as well as their distribution by disease, system affected, and geographic region.

## Results

### Study selection and characteristics of included studies

The initial search strategy identified 958 records. After removing duplicates (n = 478) and applying the eligibility criteria, 130 studies were included in the final analysis (Fig 1). These studies encompassed a wide range of methodological designs, including case reports, case series, cross-sectional studies, case-control studies, cohort studies, clinical trials, ecological analyses, and retrospective studies. Collectively, the included studies covered 25 countries. Brazil contributed the largest number of publications (n = 73), accounting for more than half of all included studies. Other countries with multiple contributions included Sri Lanka and Colombia (approximately five studies each), followed by India (approximately four studies). The United Kingdom and the United States contributed a smaller number of studies (approximately three each).

This systematic review synthesized a substantial body of evidence, including 130 studies comprising 551,574 individuals across 25 countries. Overall, 29% of assessed individuals presented at least one disability, highlighting disability as a meaningful and under-recognized outcome across neglected tropical diseases. This distribution reflects both the global scope of NTDs and the regional concentration of specific conditions, such as leprosy and Chagas disease in Latin America, or trachoma and onchocerciasis in Africa. The characteristics of the included studies are summarized in Table 1.

**Table 1:** Characteristics of the studies included in the systematic review.

#	First author, Year	Sample (n)	Female sex, n (%)	Age, mean ± SD	Geographical origin	Neglected disease
*Case report*						
1	Pires, 2020 [21]	1	0 (0)	36	Brazil	Leprosy
2	Parente, 2018 [22]	1	1 (100)	55	Brazil	Leprosy
3	Ortega, 2018 [23]	1	0 (0)	51	Colombia	Neurocysticercosis
4	Diaz-Ramirez, 2017 [24]	1	0 (0)	25	Colombia	Leprosy
5	Dametto, 2016 [25]	1	0 (0)	24	Brazil	Neurocysticercosis
6	Hernández-Beltrán, 2015 [26]	1	1 (100)	26	Colombia	Chagas disease
7	Seijo, 2014 [27]	1	0 (0)	38	Argentina	Chikungunya
8	Rigo, 2008 [28]	1	0 (0)	64	Brazil	Leishmaniasis
9	Filho, 2007 [29]	3	1 (33)	25 ± 10	Brazil	Schistosomiasis
10	Parajuli, 2020 [30]	1	0 (0)	67	Nepal	Leishmaniasis
11	Pradhan, 2020 [31]	1	1 (100)	37	Bhutan	Leishmaniasis
12	Pallangyo, 2020 [32]	1	0 (0)	38	Tanzania	Schistosomiasis
13	Dalugama, 2018 [33]	1	1 (100)	43	Sri Lanka	Dengue
14	Sadiq, 2014 [34]	1	1 (100)	50	Paquistan	Dengue
15	Chang, 2007 [35]	1	1 (100)	32	China	Dengue
16	Yan, 2006 [36]	1	0 (0)	31	Singapore	Dengue
17	Bentes, 2024 [37]	1	1 (100)	54	Brazil	Snakebite
18	Susmitha, 2024 [38]	1	0 (0)	33	India	Snakebite
*Case series*						
19	Rhee, 2014 [39]	3	3 (100)	36 ± 6	South Korea	Dengue
20	Koh, 2013 [40]	11	4 (36,4)	27.3	Singapore	Dengue
21	Espiritu, 1991 [41]	4	1 (25)	NR	USA	Leprosy
*Case-Control*						
22	Pazin-Filho, 2006 [42]	59	NR	55.0	Brazil	Chagas disease
23	Dantas, 2002 [43]	81	50 (61,7)	NR	Brazil	Chagas disease
24	Mangone, 1994 [44]	45	26 (57,7)	36.9 ± 10.5	Argentina	Chagas disease
25	Zicker, 1988 [45]	120	56 (47)	43.9	Brazil	Chagas disease
*Clinical Trial*						
26	Barda, 2017 [46]	303	168 (55,4)	6.2	Switzerland	Schistosomiasis
27	Masud, 2008 [47]	240	83 (34,5)	34	Liberia	Onchocerciasis
28	Wijesinghe, 2015 [48]	225	157 (69,7)	42.1 ± 12.4	Sri Lanka	Snakebite
29	Melese, 2005 [49]	1452	1121 (77)	50	United Kingdom	Trachoma
30	Agbenorku, 2011 [50]	38	15 (39.4)	14	Ghana	Burili Ulcer
*Cohort*						
31	Duvignaud, 2018 [51]	22	20 (89.6)	46.4 ± 12.0	England	Chikungunya
32	Ribeiro, 2015 [52]	107	55 (52,1)	NR	Brazil	Leprosy
33	Borges-Pereira, 1998 [53]	298	183 (61,4)	50.0	Brazil	Chagas disease
34	Bowman, 2001 [54]	326	208 (64)	43 ± 21	United Kingdom	Trachoma
*Cross-sectional*						
35	Bernardes, 2009 [55]	69	28 (40)	NR	Brazil	Leprosy
36	Klis, 2014 [56]	127	86 (67,7)	18.0	Netherlands	Burili Ulcer
37	Costa, 2023 [57]	24	3 (12,5)		Brazil	Leprosy
38	Bomtempo, 2023 [58]	71	38 (53,5)	46.0	Brazil	Leprosy
39	Machado, 2022 [59]	37	37 (100)	53.45 ± 7.32	Brazil	Chicungunya
40	Chaves, 2022 [60]	392	141 (35,9)	NR	Brazil	Leprosy
41	Matos, 2021 [61]	50	25 (50)	0 a > 60 anos	Brazil	Leprosy
42	D’Azevedo, 2021 [62]	43	20 (46,5)	NR	Brazil	Leprosy
43	Campos, 2020 [63]	101	66 (65,3)	NR	Brazil	Chagas disease
44	Sobral, 2020 [64]	42	6 (14,2)	NR	Brazil	Leprosy
45	Abella, 2019 [65]	94	76 (80,8)	57	Colombia	Chikungunya
46	Silva, 2019 [66]	323	NR	NR	Brazil	Leprosy
47	Robinet, 2019 [67]	14	2 (14,2)	NR	Cuba	Leprosy
48	Silva, 2018 [68]	323	134 (41,4)	37.7	Brazil	Leprosy
49	Loiola, 2018 [69]	40	18 (45)	NR	Brazil	Leprosy
50	da Silva, 2018 [70]	896	444 (49,5)	NR	Brazil	Leprosy
51	Aben-Athar, 2017 [71]	84	30 (35,7)	NR	Brazil	Leprosy
52	Basso, 2017 [72]	52	12 (23)	NR	Brazil	Leprosy
53	Belkiman-Pedro, 2018 [73]	50	9 (18)	57,06	Brazil	Leprosy
54	Del Arco, 2016 [74]	22	14 (63,6)	51 ± 10.78	Brazil	Leprosy
55	Gaudenci, 2016 [75]	32	13 (40,6)	49.3	Brazil	Leprosy
56	Monteiro, 2014 [76]	282	137 (48,5)	45.8	Brazil	Leprosy
57	Pelarigo, 2014 [77]	90	22 (24,4)	68,91 ± 7.3	Brazil	Leprosy
58	Carvalho, 2014 [78]	118	30 (25,4)	41.9	Brazil	Leprosy
59	Araújo, 2014 [79]	1770	851 (48)	57 ± 4.5	Brazil	Leprosy
60	Monteiro, 2013 [80]	282	137 (48,6)	45.8	Brazil	Leprosy
61	Carvalho, 2015 [81]	26	11 (42,3)	62	Brazil	Leprosy
62	Baldan, 2012 [82]	50	29 (58)	47 ± 7.8	Brazil	Leprosy
63	Pieri, 2014 [83]	245	116 (47,3)	46	Brazil	Leprosy
64	Silva, 2012 [84]	69	43 (62,3)	NR	Brazil	Leprosy
65	Guerrero, 2013 [85]	333	121 (36,3)	49.1	Colombia	Leprosy
66	Budel, 2011 [86]	22	10 (45)	50.2	Brazil	Leprosy
67	Seijo, 2011 [87]	13	0 (0)	38 ± 14	Argentina	St. Louis Encephalitis
68	Raposo, 2011 [88]	61	32 (52,5)	41	Brazil	Leprosy
69	de Sousa, 2011 [89]	100	46 (46)	39.2	Brazil	Leprosy
70	Nardi, 2011 [90]	384	NR	51,7 ± 15,18	Brazil	Leprosy
71	Rodini, 2010 [91]	26	11 (42,3)	51	Brazil	Leprosy
72	Silva, 2008 [92]	417	126 (30,2)	NR	Brazil	Chagas disease
73	Barbosa, 2008 [93]	69	43 (62,3)	46	Brazil	Leprosy
74	Cunha, 2009 [94]	158	22 (13,9)	>15 - < 55	Brazil	Leprosy
75	Goldschmidt, 2007 [95]	941	NR	NR	Mexico	Chlamydia trachomatis
76	Escarabel, 2008 [96]	60	12 (20)	7 ± 3.4	Brazil	Leprosy
77	Carvalho, 2000 [97]	81	43 (53)	NR	Brazil	Leprosy
78	Maradel, 1998 [98]	300	95 (31,7)	58.6	Brazil	Leprosy
79	Mol, 2023 [99]	62	39 (62,9)	NR	Netherlands	Leprosy
80	Vêncio, 1988 [100]	348	NR	NR	Brazil	Snakebite
81	Baranwal, 2020 [101]	2150	891 (41,4)	NR	India	Onchocerciasis
82	Pedrazzanie, 1985 [102]	160	NA	NR	Brazil	Leprosy
83	Olamiju, 2023 [103]	158	53 (33,5)	NR	Nigeria	Onchocerciasis
84	Getahun, 2021 [104]	778	400 (51,4)	11.3	Ethiopia	Onchocerciasis
85	Kassaw, 2020 [105]	596	292 (49)	NR	Ethiopia	Trachoma
86	Ngondi, 2006 [106]	3567	1979 (55,5)	17.6	Sudan	Trachoma
87	Frick, 2001 [107]	3064	1593 (55,2)	NR	Tanzania	Trachoma
88	Kortlang, 1996 [108]	5871	3063 (52,1)	NR	Mali	Trachoma
89	Yunia, 2023 [109]	325	119 (36,6)	53.2	United Kingdom	Leprosy
90	Bispo, 2020 [110]	42	14 (33,3)	19.5	USA	Leishmaniasis
91	Singh, 2014 [111]	302	76 (25,1)	36	India	Leprosy
92	Kayembe, 2003 [112]	750	357 (47,6)	40	Republic of Congo	Onchocerciasis
93	Dana, 1994 [113]	61	18 (29,5)	NR	USA	Leprosy
94	Rawlany, 2013 [114]	30	15 (50)	46.3	India	Leprosy
95	Reis, 2018 [115]	222	108	NR	Brazil	Leprosy
96	Jayawardana, 2018 [116]	112	49 (44)	42.8 ± 12.4	Sri Lanka	Snakebite
97	Williams, 2011 [117]	88	14 (16)	41.6 ± 11.8	Sri Lanka	Snakebite
*Ecological*						
98	Véras, 2023 [118]	1900	869 (45,7)	NR	Brazil	Leprosy
99	Pescarini, 2021 [119]	396989	177646 (44,7)	<15 anos/>15	Brazil	Leprosy
100	Albuquerque, 2020 [120]	292	151 (51,7)	NR	Brazil	Leprosy
101	Souza, 2019 [121]	4252	NR	NR	Brazil	Leprosy
102	Pereira, 2019 [122]	50673	NR	NR	Brazil	Leprosy
103	de Souza, 2017 [123]	5973	2882 (48,2)	NR	Brazil	Leprosy
104	Vieira, 2016 [124]	126	70 (55,5)	56.02 ± 15.71	Brazil	Leprosy
105	Oliveira, 2010 [125]	542	278 (51,2)	42.5 ± 17.2	Brazil	Leprosy
106	Lana, 2008 [126]	1456	711 (48,8)	NR	Brazil	Leprosy
107	Sobrinho, 2007 [127]	99	32 (32,3)	50 ± 8.8	Brazil	Leprosy
108	Duarte, 2007 [128]	37	16 (43,2)	42 ± 3.3	Brazil	Leprosy
109	de Souza, 2006 [129]	187	78 (42.2)	NR	Brazil	Chagas disease
110	Al Faran, 2994 [130]	19	NR	NR	Saudi Arabia	Trachoma
111	Petrela, 2010 [131]	1210	508 (42)	8 ± 3.1	USA	Leishmaniasis
112	Jayawardana, 2016 [132]	816	326 (40)	42.7 ± 16.3	Sri Lanka	Snakebite
113	Aglanu, 2022 [133]	193	98 (50,8)	37 ± 9.7	Gana	Snakebite
*Prospective*						
114	Suman, 2017 [134]	107	49 (45,7)	57	Brazil	Chagas disease
*Retrospective*						
115	Bentes, 2021 [135]	65	43 (66,1)	NR	Brazil	Leprosy
116	Souza, 2019 [136]	42227	NR	NR	Brazil	Leprosy
117	Santana, 2018 [137]	414	211 (51)	NR	Brazil	Leprosy
118	Benedicto, 2018 [138]	100	66 (66)	NR	Brazil	Leprosy
119	Queirós, 2016 [139]	475	242 (51,8)	45,2	Brazil	Leprosy
120	Galan, 2016 [140]	11	2 (18,1)	NR	Brazil	Leprosy
121	de Oliveira, 2013 [141]	494	227 (46)	NR	Brazil	Leprosy
122	Kil, 2012 [142]	318	NR	38	Brazil	Leprosy
123	Julio, 2010 [143]	212	NR	46.6 ± 16.2	Brazil	Leprosy
124	Araújo, 2006 [144]	10	3 (30)	7.3	Brazil	Schistosomiasis
125	Mercado, 2018 [145]	13	3 (23)	NR	Netherlands	Chikungunya
126	Abiose, 1994 [146]	6831	NR	NR	Nigeria	Onchocerciasis
127	Akogun, 1992 [147]	2876	1314 (45,6)	NR	Nigeria	Onchocerciasis
128	Newland, 1991 [148]	800	288 (36,1)	NR	Africa	Onchocerciasis
129	Brenes-Chacon, 2020 [149]	74	22 (29,7)	NR	Costa Rica	Snakebite
130	Silva, 2021 [150]	307	65 (21)	38.6 ± 14.5	Brazil	Snakebite

NR: Not reported.

**Fig 2:**
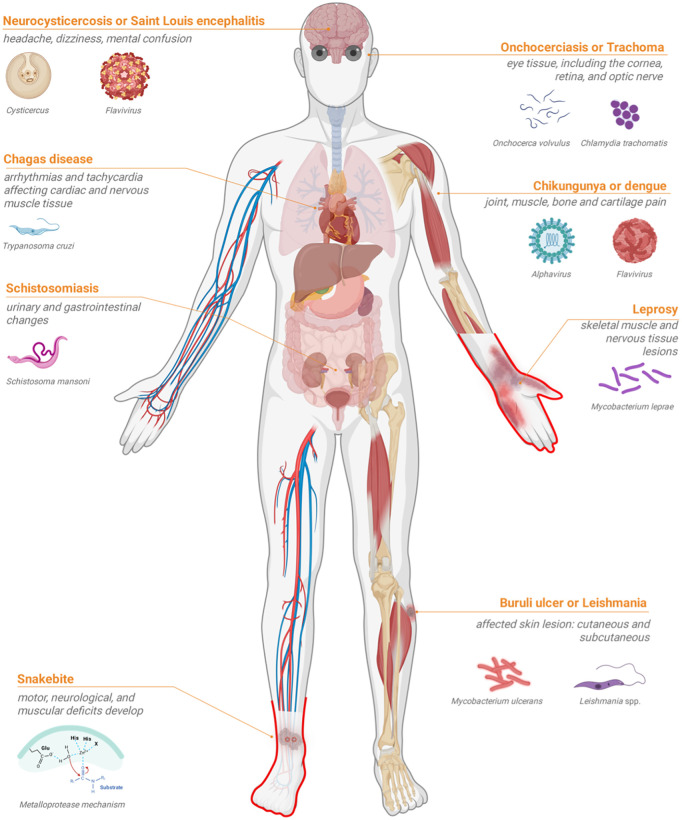
Systems and organs affected by disabilities related to neglected tropical diseases. Distribution of sequelae reported in the included studies, grouped by affected system (neurological, musculoskeletal, visual, cardiovascular, gastrointestinal, psychosocial, and others).

The country with the highest number of studies of leprosy was Brazil, with a total of 60 studies, followed by snakebites (n = 10) and Chagas Disease (n = 8). However, the incidence of other NTDs was unevenly distributed, with wide distribution in countries in Africa, Asia and the Americas, and low or absent incidence in Europe and Oceania, respectively (Fig 2).

### Demographic profile of study populations

The included studies encompassed a total of 551,574 individuals. Sample sizes ranged from single patient case reports to large-scale ecological studies with more than 390,000 participants. The proportion of female participants varied widely, from 12.5% to 100%, and mean age ranged from 6.2 years in a schistosomiasis trial to 68.9 years in a cross-sectional leprosy study conducted in Brazil. Despite this diversity, demographic data such as age and sex were frequently absent, particularly in older publications, limiting the possibility of conducting detailed subgroup analyses by sex or age group.

### Reported comorbidities

Comorbidities were infrequently and inconsistently reported across studies. Among the reported conditions, respiratory diseases were the most frequent (0.2%, n = 1,019), followed by heart diseases (0.1%, n = 540). Mental health conditions, including anxiety and depression, were reported in 0.05% (n = 295) of individuals. Other comorbidities included hypertension (0.03%, n = 180), diabetes (0.02%, n = 149), and obesity (0.01%, n = 87). Despite the low reported frequencies, these findings likely reflect substantial underreporting across studies (Table 2).

**Table 2:** Comorbidities and sequelae reported by the populations evaluated in the included studies.

Total evaluated	N = 551574
*Reported comorbidities*	
Respiratory diseases (%, n)	0,2 (n = 1019)
Heart diseases (%, n)	0,1 (n = 540)
Anxiety/Depression (%, n)	0,05 (n = 295)
Hypertension (%, n)	0,03 (n = 180)
Diabetes (%, n)	0,02 (n = 149)
Obesity (%, n)	0,01 (n = 87)
*Reported Sequelae*	
Physical/motor disabilities (%, n)	24,6 (n = 135683)
Visual deficits (%, n)	1,81 (n = 10007)
Skin problems (%, n)	0,33 (n = 1816)
Gastrointestinal deficits (%, n)	0,25 (n = 1379)
Fever (%, n)	0,24 (n = 1319)
Appetite changes (%, n)	0,19 (n = 1034)
Abdominal pain (%, n)	0,18 (n = 985)
Nerve injuries (%, n)	0,17 (n = 964)
Motor dysfunctions (%, n)	0,15 (n = 803)
Fatigue (%, n)	0,12 (n = 667)
Paresthesia (%, n)	0,10 (n = 538)
Dyspnea (%, n)	0,09 (n = 437)
Cough (%, n)	0,07 (n = 373)
Muscle pain (%, n)	0,06 (n = 304)
Arrhythmias/tachycardias (%, n)	0,05 (n = 273)
Joint pain (%, n)	0,04 (n = 218)
Urinary changes (%, n)	0,03 (n = 159)
Depression (%, n)	0,03 (n = 158)
Psychological disorders (%, n)	0,02 (n = 135)
Emotional impacts in work (%, n)	0,02 (n = 127)
Headache (%, n)	0,02 (n = 112)
Limb edema (%, n)	0,02 (n = 110)
Hypertension (%, n)	0,02 (n = 85)
Chest pain (%, n)	0,01 (n = 66)
Dysphagia (%, n)	0,01 (n = 57)
Hearing problems (%, n)	0,01 (n = 45)
Emotional difficulties in family life (%, n)	0,01 (n = 42)
Muscle weakness (%, n)	0,01 (n = 40)
Deficiency in social life (%, n)	0,01 (n = 37)
Anxiety (%, n)	0,01 (n = 36)
Mental confusion (%, n)	0,004 (n = 26)
Sleep problems (%, n)	0,004 (n = 23)
Memory problems (%, n)	0,003 (n = 21)
Cognitive deficits (%, n)	0,003 (n = 18)
Voice/speech changes (%, n)	0,001 (n = 7)

### Prevalence and types of disabilities

The synthesis of the included studies revealed that long-term disabilities are a major consequence of NTDs. Overall, 29% (n = 158,104) of all evaluated individuals presented with at least one form of disability.

A wide range of additional sequelae were reported with lower prevalence. These included fever (0.24%, n = 1,319), appetite changes (0.19%, n = 1,034), abdominal pain (0.18%, n = 985), motor dysfunctions (0.15%, n = 803), and fatigue (0.12%, n = 667). Respiratory and cardiovascular-related symptoms included dyspnea (0.09%, n = 437), cough (0.07%, n = 373), and arrhythmias or tachycardia (0.05%, n = 273). Physical and motor disability predominated (24.6%, n = 135,683), followed by visual disability (1.81%, n = 10,007). Cardiovascular, neurological, and psychosocial sequelae were also reported, broadening the spectrum of disability outcomes associated with neglected tropical diseases, followed by skin-related conditions (0.33%, n = 1,816).

Other sequelae encompassed a broad spectrum of manifestations. Neurological outcomes included nerve injuries (0.17%, n = 964), paresthesia (0.10%, n = 538), and cognitive or memory deficits (<0.01%). Psychological and social impairments were also present, such as depression (0.03%, n = 158), psychological disorders (0.02%, n = 135), and family or work-related emotional difficulties (0.01–0.02%). Functional and systemic symptoms were frequently observed, including gastrointestinal complaints (0.25%, n = 1,379), and muscle pain (0.06%, n = 304).

### Disease-specific distribution of sequelae

**Fig 3:**
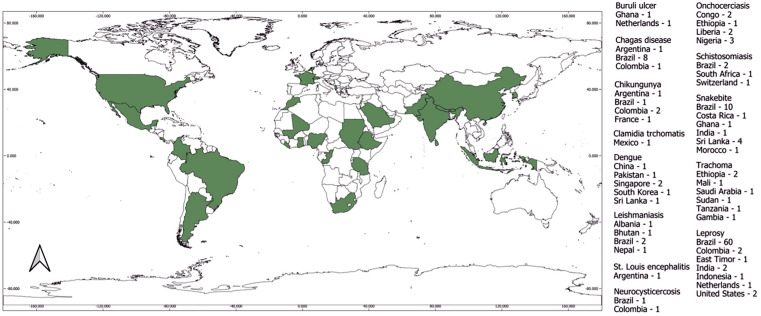
Map showing the location of study samples. Distribution of studies reporting sequelae included in systematic review.

The distribution of disabilities varied considerably by disease (Fig 3). Leprosy accounted for most physical and motor impairments, consistent with its well-documented impact on the peripheral nervous system. Trachoma was the leading cause of visual impairment, followed by onchocerciasis. Cardiovascular impairments were overwhelmingly associated with Chagas disease, where chronic cardiomyopathy remains the hallmark condition. Snakebite envenoming was frequently reported as a cause of local tissue damage and persistent motor dysfunction, although prevalence estimates varied substantially across studies. Neurological sequelae were prominent in diseases such as neurocysticercosis and human African trypanosomiasis, manifesting as seizures, cognitive decline, or other forms of neutralizability.

### Regional distribution of sequelae

Regional patterns were also evident. In Latin America, leprosy and Chagas disease dominated the profile of disabilities, while in Africa, trachoma and onchocerciasis accounted for most of the reported impairments. In Asia, studies more frequently reported snakebite-related disabilities, as well as complications from leprosy and dengue. However, some WHO-listed NTDs, such as yaws, dracunculiasis, and scabies, were rarely, or not represented, in the included studies, highlighting persistent gaps in evidence in this manner.

### Risk of bias and reporting limitations

The methodological quality of the included studies was highly heterogeneous. In general, more recent publications showed greater adherence to reporting standards, while older studies frequently failed to provide essential demographic information such as age and sex. Using the MINORS tool [18,19], most cross-sectional and cohort studies scored between 12 and 16 out of a maximum of 16 points, reflecting some incomplete reporting of endpoints, sample representativeness, and follow-up details. Case reports and series, assessed through the Critical Appraisal Checklist [19], often lacked clear descriptions of clinical pathways and standardized definitions of disability. The clinical trials assessed using the Jadad scale [20], demonstrated adequate methodological quality.

## Discussion

This systematic review provides a comprehensive overview of the disabilities associated with NTDs, synthesizing data from 130 studies and encompassing over half a million individuals. The findings reveal that physical and motor disabilities are the most frequently reported sequelae, affecting a substantial portion of the studied population. This underscores the lasting impact of NTDs on the functional capacity and quality of life of affected individuals. The high prevalence of these disabilities highlights a critical gap in the management of NTDs, which has historically focused more on incidence, mortality and transmission control than on long-term morbidity and rehabilitation [11]. This review synthesizes evidence from a large and heterogeneous body of literature and reinforces that disability represents a substantial and under-recognized outcome across neglected tropical diseases. The descriptive estimate that 29% of individuals presented at least one disability highlights the magnitude of long-term functional consequences beyond infection control.

The predominance of physical and motor disabilities (24.6%) in our findings aligns with the well-documented impact of diseases like leprosy, which was the most frequently addressed NTD in the included studies. Leprosy is known to cause significant nerve damage, leading to sensory loss, muscle weakness, and visible deformities, which severely impair motor function [151–153]. Our review included a large number of studies from Brazil, a country with a high burden of leprosy, which may have contributed to the high prevalence of physical disabilities observed. A large cohort study in Brazil, for instance, found that 27% of new leprosy cases presented with some degree of physical disability (Grades 1 or 2), with multibacillary forms of the disease being associated with an over 8-fold increased risk of Grade 2 disability [11,154]. This emphasizes that beyond the infectious process itself, the resulting chronic disabilities constitute a major public health challenge, perpetuating cycles of poverty and social stigma [11].

Visual impairment was the second most common disability identified in this review (1.81%). This is largely attributable to diseases like trachoma and onchocerciasis. Trachoma, caused by Chlamydia trachomatis, is the world’s leading infectious cause of blindness. Repeated infections lead to trachomatous trichiasis (TT), where the eyelashes turn inward and scrape the cornea, eventually causing corneal opacity (CO) and irreversible blindness [155]. A recent meta-analysis estimated that, globally, over 1.9 million people are blind or have severe to moderate vision impairment due to trachoma [155]. Although the prevalence of trachomatous blindness has been decreasing due to successful control strategies, it remains a significant problem in many endemic areas, particularly in Africa [155]. The impact of visual impairment extends beyond the loss of sight, affecting individuals’ ability to work, their social participation, and their overall quality of life.

Cardiovascular complications, although reported less frequently in the included studies, are a hallmark of Chagas disease, another of the most studied NTDs in this review. Chronic Chagas cardiomyopathy is a severe and often fatal consequence of infection with Trypanosoma cruzi, affecting up to 30% of chronically infected individuals [156]. It leads to a range of cardiovascular issues, including heart failure, arrhythmias, stroke, and sudden death. The pathogenesis is complex, involving a parasite-driven immune response, direct tissue damage, and microvascular derangements that result in progressive myocardial fibrosis and dysfunction [156]. The burden of chagas disease is substantial, not only in Latin America but also in non-endemic countries due to migration [156]. The disabilities resulting from cardiac complications, such as fatigue, dyspnea, and reduced functional capacity, severely limit the daily activities of patients and impose a significant economic burden on health systems.

This review also identified a wide range of other sequelae, including neurological, psychological, and social impacts, which are often under-recognized. Neurological impairments, for example, are not limited to the peripheral nerve damage in leprosy but are also a feature of diseases like neurocysticercosis and human African trypanosomiasis [11,157]. Furthermore, the social stigma and mental health consequences associated with the disfiguring and disabling nature of many NTDs are profound [11,157]. Depression, anxiety, and social exclusion are common among people affected by NTDs, further exacerbating their suffering and hindering their access to care and participation in society [153].

The methodological heterogeneity observed in the studies included in this review poses a significant challenge to evidence synthesis and policymaking. The lack of standardized disability definitions and the variety of assessment tools used make it difficult to compare data across different studies and regions, likely resulting in an underestimation of the true burden of disability. In response to this gap, efforts have been made to develop and validate cross-NTD morbidity and disability assessment toolkits, such as the “cross-NTD toolkit” [158].

Despite the substantial burden of long-term disabilities identified in this review, access to rehabilitation services for individuals affected by NTDs remains profoundly limited and inadequately integrated into health systems. In most endemic settings, care models continue to prioritize acute disease control, case detection, and pharmacological treatment, while long-term functional outcomes and rehabilitation needs receive minimal clinical attention [159]. Consequently, chronic sequelae, such as motor impairment, sensory loss, visual deficits, cognitive limitations, and psychosocial consequences, are frequently under-recognized or insufficiently addressed by physicians and healthcare teams managing NTDs. This gap often results in delayed diagnosis of disability, low referral rates to rehabilitation services, and fragmented or absent continuity of care. These shortcomings directly contradict the principles of the WHO Rehabilitation 2030 initiative and Universal Health Coverage, which emphasize rehabilitation as an essential health service across the life course. Furthermore, the scarcity of rehabilitation professionals, assistive technologies, and community-based rehabilitation programs, particularly in low- and middle-income countries where NTDs have higher incidence, exacerbates structural inequities in access. Financial barriers, geographic isolation, limited workforce capacity, and insufficient policy prioritization further restrict service availability. Addressing these gaps is essential to reduce avoidable disability, improve functional independence, and ensure that NTD control strategies move beyond infection elimination toward truly patient-centered and disability-inclusive care.

This systematic review has several limitations. First, the included studies were highly heterogeneous in terms of design, methodology, disability definitions, and assessment instruments, which limited comparability and precluded meta-analysis. Second, there was substantial underreporting of disabilities, and definitions varied widely across studies, hampering comparison of findings. The frequent use of “not reported” (NR) for key demographic data, such as age and sex, particularly in older studies, also limited subgroup analyses. In addition, the evidence base was dominated by leprosy and by studies from a limited number of countries, particularly Brazil, which may restrict generalizability to other neglected tropical diseases and settings. Many included studies were observational and facility-based, raising the possibility of selection bias toward more severe cases, as individuals with milder or asymptomatic conditions are less likely to seek care or be captured in clinical settings. Finally, limited longitudinal data, underreporting of psychosocial outcomes, and potential publication bias may have influenced the overall estimates.

## Conclusions

The findings of this systematic review demonstrate that disabilities are a major and often neglected consequence of NTDs. The high prevalence of physical, visual, and other impairments highlights the urgent need to integrate rehabilitation and disability-inclusive development into NTD control programs. A paradigm shift is needed, moving beyond a purely biomedical approach to a more holistic one that addresses the long-term physical, psychological, and social needs of people affected by NTDs, alongside with reintegration into daily-life, social and work-related activities. Future research should focus on developing and evaluating effective and scalable rehabilitation interventions, as well as on improving the standardization of disability assessment and reporting in NTD studies.
