# Supplementary material for: Disability as a neglected outcome of neglected tropical diseases: A systematic review
Source: PLoS Negl Trop Dis. 2026 Jul 1;20(7):e0014480. doi: 10.1371/journal.pntd.0014480 (PMC13345467; doi:10.1371/journal.pntd.0014480)
Supplement: Attachment — Submitted filename: Response to Reviewers Caio-2.docx [file pntd.0014480.s001.docx]

**Response to Reviewers**

Manuscript: *Disability as a neglected outcome of neglected tropical diseases: a systematic review*

Manuscript ID: PNTD-D-26-00064

Dear Editor and Reviewers,

We sincerely thank the reviewers for their careful evaluation and constructive comments. We have revised the manuscript extensively to improve methodological transparency, PRISMA compliance, data availability, and clarity of interpretation. All changes have been incorporated in the revised manuscript and are detailed below.

Please provide an Author Summary. This should appear in your manuscript between the Abstract (if applicable) and the Introduction and should be 150-200 words long. The aim should be to make your findings accessible to a wide audience that includes both scientists and non-scientists.

**Response:** We thank the editor for this request. We have added the following Author Summary between the Abstract and Introduction.

**Author Summary**

Neglected tropical diseases (NTDs) affect more than one billion people worldwide, primarily in low-resource settings. While global control efforts have focused on reducing transmission and mortality, the long-term disabilities caused by these diseases remain poorly described and rarely incorporated into public health strategies. In this systematic review, we analyzed 130 studies including 551,574 individuals across 25 countries to identify the types and frequency of disabilities associated with NTDs. Overall, 29% of individuals experienced at least one disability. Physical and motor impairments were the most common, largely related to leprosy, followed by visual impairment associated with trachoma and onchocerciasis. Cardiovascular, neurological, and psychosocial consequences were also reported. These findings highlight that disability is a substantial and underrecognized outcome of NTDs, extending beyond infection control. The evidence also revealed considerable heterogeneity in how disability is defined and measured, as well as underreporting across diseases. Our results emphasize the need to integrate disability assessment, rehabilitation services, and disability-inclusive policies into NTD programs. Addressing long-term functional outcomes is essential to improve quality of life and to achieve the goals of Universal Health Coverage and the WHO NTD Roadmap.

**Reviewer #1**

Reviewer #1: The hypothesis and the objectives are clear and appropriate to this systematic review. The study design is interesting, and several tools for systematic reviews are mentioned. The sample is large (130 studies found). Given the number of publications, this systematic review could deserve a meta-analysis. In addition, being a systematic review following PRISMA (2020) guidelines, some information is missing. Namely, the authors did not formulate the question of research with the PICO items.

**Response:** We agree and have now explicitly defined the research question using a PICO-adapted framework appropriate for systematic reviews of heterogeneous designs, following PRISMA 2020 recommendations.

Added to Methods:

The research question was structured using a PICO-adapted framework appropriate for descriptive systematic reviews, in which the population comprised individuals diagnosed with WHO-listed neglected tropical diseases; the exposure corresponded to the presence of long-term disability or sequelae; no comparison group was defined due to the descriptive nature of the synthesis; and the outcomes included the type and prevalence of disability.

Reviewer #1: Moreover, the number of the protocol register in PROSPERO is not presented (Item 24 of PRISMA checklist), and it is a requirement for a true systematic review. In the search strategy and data sources, it is stated that no restrictions were applied for language and publication date, and information about supplementary materials is given.

**Response:** We thank the reviewer. The PROSPERO registration has now been included:

PROSPERO registration: CRD420261351709

Reviewer #1: Regarding study selection, the number of studies in each step presented in the flow chart should be revised, since in the screening step, if 179 were excluded from the 480 records screened, it remains 301 (not 310). And excluded 180 from these, only 121 remain in the final. In the flow chart (Figure 1), the number of duplicate records is 478, but at the beginning of the results, it is mentioned 345 duplicates. So, it’s important to verify all the numbers and replace where it is wrong.

**Response:** We carefully rechecked all counts and corrected:

duplicates removed

screening exclusions

final included studies

The PRISMA flow diagram has been revised accordingly.

Reviewer #1: Table 1, in the second column, presents “Author, Year”, but the studies have more than one author. The appropriate references should be indicated, for example, using “et al.” if more than three authors.

**Response:** Thank you for the suggestion. We agree that the use of “et al.” is appropriate in formal citations. However, in Table 1, studies are presented for identification purposes only, and the use of “first author + year” is a widely adopted approach in systematic reviews to improve readability.

To better reflect this rationale and avoid potential ambiguity, we have revised the column heading from “Author, Year” to “First author, Year”.

Since full references are provided in the reference list, we have retained this format. Nevertheless, we would be happy to further revise it if required by the journal’s editorial style.

Reviewer #1: In addition, some abbreviations need to be described before their use (for example, “NR” on page 11).

**Response:** All abbreviations now defined at first use.

### **Reviewer #2**

### Screening was performed by two reviewers with adjudication by a third reviewer; please clarify whether data extraction was also performed in duplicate (or how errors were minimized), and how disagreements were handled. Please consider reporting agreement for title/abstract screening and full-text eligibility assessment (e.g., Cohen’s kappa, weighted kappa, or percent agreement) and clarify whether data extraction was also conducted in duplicate. This would improve transparency and confidence in the review process and align with good reporting practices (e.g., PRISMA). If agreement metrics were not calculated, please provide a brief justification and describe any calibration/training steps and quality-control procedures used to minimize selection and extraction errors.

### **Response:** We thank the reviewer for this important suggestion. We have clarified the screening and data extraction procedures in the Methods section to improve transparency and align with PRISMA 2020 recommendations.

Title/abstract screening and full-text eligibility assessment were independently performed by two reviewers, with disagreements resolved by a third reviewer. Data extraction was also conducted in duplicate by two independent reviewers using a standardized and piloted extraction form. Any discrepancies were resolved through discussion and, when necessary, adjudication by a third reviewer. Before formal screening, a calibration exercise was conducted using a random sample of studies to ensure consistency in eligibility criteria and data extraction. Because agreement metrics (e.g., Cohen’s kappa) were not prospectively calculated, we have clarified that consensus procedures, calibration training, and duplicate extraction were implemented to minimize selection and extraction errors, consistent with recommended systematic review practices (PRISMA 2020).

This information has now been added to the Methods section.

### Reviewer #2: The manuscript uses MINORS for observational studies, CARE for case reports/series, and the Jadad scale for randomized controlled trials. This may be acceptable; however: MINORS is not currently the most commonly used instrument for observational studies in systematic reviews (many journals prefer ROBINS-I or the Newcastle–Ottawa Scale, depending on the study design). The Results section reports overall scores (e.g., “18–24/24”) but does not clearly show how risk of bias influenced the interpretation of the findings.

### **Response:** We thank the reviewer for this thoughtful comment. We have strengthened the justification for the choice of methodological quality assessment tools and improved the integration of risk-of-bias results into the interpretation of findings.

We selected the MINORS instrument for observational studies because the included evidence base consisted predominantly of non-randomized and descriptive clinical studies, for which MINORS has been widely used in systematic reviews evaluating heterogeneous observational designs. CARE guidelines were applied to case reports and case series to ensure structured methodological assessment, and the Jadad scale was used for randomized controlled trials. This approach allowed methodological appraisal tailored to each study design.

To improve transparency, we have now:

(i) added a study-by-study risk-of-bias table stratified by study design in the Supplementary Material;

(ii) clarified in the Results section how methodological quality influenced interpretation, emphasizing that conclusions were primarily driven by studies with moderate-to-high quality scores;

(iii) conducted a sensitivity analysis stratifying findings by study design and methodological quality. This analysis showed that the overall pattern of disability outcomes remained consistent, indicating that the main conclusions were not driven by lower-quality studies.

Reviewer #2: Was the review protocol registered in PROSPERO?

**Response:** Yes. The review protocol was registered in PROSPERO prior to data extraction. The registration number is:
PROSPERO CRD420261351709. This information has now been added to the Abstract and Methods sections.

Reviewer #2: The review synthesizes a substantial body of literature (130 studies, 551,574 individuals, 25 countries), which supports the central argument that disability is a meaningful and under-recognized outcome across NTDs.
Presenting a descriptive estimate that 29% of assessed individuals had “at least one disability” is an effective headline for communicating relevance.
The Results highlight that physical/motor disability predominates (24.6%), followed by visual disability (1.81%), and they also mention cardiovascular, neurological, and psychosocial sequelae, which helps broaden the reader’s view beyond a single outcome type. The manuscript explicitly notes heterogeneity and underreporting, which is essential framing for an evidence base spanning multiple diseases and instruments.

The review has several important limitations. First, there is substantial heterogeneity in how “disability” and “sequelae” are defined and measured across studies (different instruments, domains, and thresholds), which reduces comparability and limits the strength of quantitative synthesis. Second, the evidence base is dominated by leprosy and by a small number of countries/regions (notably Brazil), which may constrain generalizability to other NTDs and epidemiological settings. This systematic review addresses an important and under-recognized topic for PLOS NTDs: disability as a long-term outcome of NTDs. The search appears comprehensive (multiple databases, no language/date restrictions) and the manuscript follows PRISMA 2020, with a sizeable included evidence base.

### **Response:** We thank the reviewer for the careful evaluation and constructive comments. We agree that the review synthesizes a substantial body of literature and highlights disability as a meaningful and under-recognized outcome across neglected tropical diseases (NTDs). To improve clarity, we revised the Results section to more explicitly report the overall sample (130 studies, 551,574 individuals, 25 countries), the descriptive estimate that 29% of individuals presented at least one disability, and the distribution of disability types, emphasizing the predominance of physical/motor disability (24.6%), followed by visual disability (1.81%), as well as cardiovascular, neurological, and psychosocial sequelae.

We also strengthened the Discussion to emphasize heterogeneity in disability definitions, assessment tools, and reporting across diseases and study designs. The limitations section was revised to explicitly acknowledge the predominance of leprosy studies, geographic concentration in certain regions (notably Brazil), the observational and facility-based nature of many studies, potential selection bias toward more severe cases, underreporting of psychosocial outcomes, limited longitudinal evidence, and the absence of meta-analysis due to methodological heterogeneity. These revisions provide a more cautious interpretation of the descriptive prevalence estimate.
