# Supplementary material for: Disability as a neglected outcome of neglected tropical diseases: A systematic review
Source: PLoS Negl Trop Dis. 2026 Jul 1;20(7):e0014480. doi: 10.1371/journal.pntd.0014480 (PMC13345467; doi:10.1371/journal.pntd.0014480)
Supplement: Attachment — Submitted filename: Manuscrito_Caio_01-04-2026_Rev2.docx [file pntd.0014480.s002.docx]

**Title: Disability as a neglected outcome of neglected tropical diseases: a systematic review**

**Authors:** Caio Cesar Leiva Bastos Barrionuevo^1,4 *^, Jefferson da Silva Valente^1*^, Bernardo Maia da Silva^1^, Cassia da Luz Goulart^4^, Alex Maciel^1^, Aldair Darlan Santos-de-Araújo^2^, Camila Miriam Suemi Sato Barros do Amaral^5,^ Eduardo Fernandes da Silva Junior^1^, Stephanie Vitória Alves dos Santos^1^, Erika Gomes^5^, Nadia Cubas-Veja^6^, Guilherme Peixoto Tinoco Arêas^3^, Fernando Almeida-Val^1,3,4,7^

**Affiliations:**

^1^ Universidade do Estado do Amazonas (UEA), Manaus – AM, Brazil.

^2^ Universidade Federal de São Carlos (UFSCar), São Carlos – SP, Brazil.

^3^ Universidade Federal do Amazonas (UFAM), Manaus – AM, Brazil.

^4^ Fundação de Medicina Tropical Dr Heitor Vieira Dourado (FMT-HVD), Manaus – AM, Brazil.

^5^ Fundação de Hematologia e Hemoterapia do Amazonas (FHEMOAM), Manaus – AM, Brazil.

^6^Fundación Cristianos Asistiendo a Quemados (CRISAQ), Tegucigalpa, Honduras

^7^Universidade Nilton Lins (UNL), Manaus – AM, Brazil

* These authors contributed equally

**Corresponding author:**

Fernando Almeida-Val. Fundação de Medicina Tropical Doutor Heitor Vieira Dourado, Av. Pedro Teixeira, 25 - Dom Pedro, Manaus - AM, 69040-000, Manaus – AM, Brazil. E-mail: ffaval@gmail.com

**ABSTRACT**

**Background:** Neglected tropical diseases (NTDs) affect more than one billion people worldwide, predominantly in low-resource settings. While substantial progress has been made in reducing transmission and mortality, the long-term disabilities resulting from NTDs remain insufficiently recognized and inadequately addressed within control programs. **Objective:** To identify, describe, and systematize the types of disabilities reported among individuals affected by NTDs, providing a comprehensive overview of the magnitude and diversity of long-term functional impairments associated with these conditions. **Methods:** This systematic review was conducted in accordance with PRISMA 2020 guidelines. A comprehensive search was performed in PubMed, Cochrane Central, Embase, CINAHL, LILACS, and Web of Science databases, without restrictions on language or publication date. Studies reporting long-term disabilities or sequelae in individuals diagnosed with WHO-listed NTDs were included. Data on study characteristics, population demographics, and disability types were extracted and synthesized descriptively. Methodological quality was assessed using design-specific tools (MINORS, CARE checklist, and Jadad scale). **Results:** The initial search identified 958 records across databases. After removal of duplicates (n = 478), 480 records were screened, of which 310 were assessed for full-text eligibility. Following the application of inclusion and exclusion criteria, 130 studies were included in the final analysis, encompassing 551,574 individuals from 25 countries. Overall, 29% (n = 158,104) of evaluated individuals presented with at least one form of disability. Physical and motor impairments were the most prevalent (24.6%, n = 135,683), predominantly associated with leprosy-related neuropathic and musculoskeletal sequelae. Visual impairment was the second most frequent disability (1.81%, n = 10,007), largely attributable to trachoma and onchocerciasis. Cardiovascular, neurological, psychological, and social impairments were also reported. Considerable heterogeneity and underreporting of disability outcomes were observed across studies. **Conclusions:** Disability represents a substantial and underappreciated outcome of NTDs, extending far beyond infection-related morbidity. The high burden of long-term physical, visual, and other functional impairments underscores the urgent need to integrate disability assessment, rehabilitation services, and disability-inclusive development into NTD control strategies. Addressing these gaps is essential to advance patient-centered care and achieve the broader goals of the WHO NTD Roadmap and Universal Health Coverage.

**Keywords:** Neglected tropical diseases, disability, long-term sequelae, rehabilitation, global health

**Author Summary**

Neglected tropical diseases (NTDs) affect more than one billion people worldwide, primarily in low-resource settings. While global control efforts have focused on reducing transmission and mortality, the long-term disabilities caused by these diseases remain poorly described and rarely incorporated into public health strategies. In this systematic review, we analyzed 130 studies including 551,574 individuals across 25 countries to identify the types and frequency of disabilities associated with NTDs. Overall, 29% of individuals experienced at least one disability. Physical and motor impairments were the most common, largely related to leprosy, followed by visual impairment associated with trachoma and onchocerciasis. Cardiovascular, neurological, and psychosocial consequences were also reported. These findings highlight that disability is a substantial and underrecognized outcome of NTDs, extending beyond infection control. The evidence also revealed considerable heterogeneity in how disability is defined and measured, as well as underreporting across diseases. Our results emphasize the need to integrate disability assessment, rehabilitation services, and disability-inclusive policies into NTD programs. Addressing long-term functional outcomes is essential to improve quality of life and to achieve the goals of Universal Health Coverage and the WHO NTD Roadmap.

**INTRODUCTION**

It is estimated that more than one billion people are affected by neglected tropical diseases (NTDs), which are prevalent in tropical and subtropical regions and are often associated with poverty and poor sanitation infrastructure (1,2). Each year, approximately 1.5 billion people require preventive or therapeutic interventions, underscoring the persistence of these diseases as a major global public health challenge and the need for coordinated action among governments, international organizations, and communities (1–4). In response, the World Health Organization (WHO) developed the NTDs 2021–2030 roadmap, proposing a shift from isolated vertical programs toward integrated and transversal strategies aimed at reducing by 90% the number of people requiring treatment, eradicating diseases, and eliminating at least one NTD in 100 countries by 2030 (5). These strategies emphasize coordinated interventions such as vector control and improvements in water, sanitation, and hygiene (5).

NTDs are characterized by marked clinical heterogeneity, with manifestations ranging from acute to chronic and from localized to systemic conditions (6,7). Their frequently silent or insidious progression, combined with diverse signs and symptoms, hampers early diagnosis and increases morbidity, particularly among vulnerable populations with limited access to healthcare services(8–11). Beyond their immediate clinical effects, NTDs are strongly associated with chronic disabilities and social stigmatization, leading to discrimination, social exclusion, and substantial economic and social consequences (8,12–17).

Physical and functional disabilities represent some of the most significant long-term consequences of NTDs. These impairments can compromise activities of daily living, reduce mobility and work capacity, and result in persistent disabling symptoms that undermine individual autonomy (6,7). Often underestimated, such disabilities not only perpetuate cycles of poverty and exclusion but also impose a considerable burden on health systems, particularly in low-resource settings where access to rehabilitation services is limited (6,7). Delayed diagnosis and inadequate treatment further exacerbate functional decline, increasing the need for long-term care. In this context, effective NTD control strategies, such as elimination programs, mass drug administration, and educational initiatives, depend on a robust understanding of regional and local dynamics, identification of at-risk populations, continuous monitoring of health indicators, and the availability of high-quality scientific evidence to inform policy and practice.

Despite this burden, disability assessment planning and structured rehabilitation strategies remain largely absent from NTD control programs. The persistence of physical and functional disabilities continues to limit affected individuals’ access to education, employment, income generation, and community participation. This challenge is compounded by scarce and fragmented data, underreporting of disabilities, and substantial methodological heterogeneity across studies, which hinder the consolidation of evidence. Therefore, this systematic review hypothesizes that disabilities associated with NTDs have been described in a fragmented and poorly standardized manner in the literature, limiting the understanding of their magnitude and complexity. The objective of this review is to identify and describe the types of disabilities reported among individuals affected by NTDs in peer-reviewed published literature, contributing to a clearer and more comprehensive understanding of the scope of this problem worldwide.

**METHODS**

**Study design and reporting standards**

This systematic review was conducted and reported in accordance with the Preferred Reporting Items for Systematic Reviews and Meta-Analyses (PRISMA 2020) guidelines (18). The objective was to identify and synthesize evidence on long-term disabilities and sequelae associated with NTDs, regardless of study design, geographic setting, language, or year of publication. The review protocol was registered in the International Prospective Register of Systematic Reviews (PROSPERO under the registration number CRD420261351709).

**Research question**

The research question was structured using a PICO-adapted framework appropriate for descriptive systematic reviews, in which the population comprised individuals diagnosed with WHO-listed neglected tropical diseases; the exposure corresponded to the presence of long-term disability or sequelae; no comparison group was defined due to the descriptive nature of the synthesis; and the outcomes included the type and prevalence of disability.

**Search strategy and data sources**

A comprehensive literature search was performed in May 2025 across the following electronic databases: PubMed (MEDLINE), Cochrane Central Register of Controlled Trials (CENTRAL), Embase (Elsevier), CINAHL, Latin American and Caribbean Health Sciences Literature (LILACS), and Web of Science (Clarivate Analytics). The search strategy combined Medical Subject Headings (MeSH) and entry terms related to NTDs as defined by the WHO (1), together with terms related to disability, sequelae, functional impairment, and loss of function, using Boolean operators (AND, OR, NOT). No restrictions were applied regarding language or publication date. To ensure completeness, backward and forward citation searches were conducted by screening the reference lists of included studies and relevant systematic reviews. The full search strategy, including all MeSH terms and entry terms for WHO-listed NTDs, is provided in the Supplementary Material.

Title and abstract screening and full-text eligibility assessment were independently performed by two reviewers. Disagreements were resolved by discussion and, when necessary, adjudication by a third reviewer. Prior to screening, a calibration exercise was conducted using a random sample of studies to ensure consistency in the application of eligibility criteria. Agreement metrics (e.g., Cohen’s kappa) were not prospectively calculated. However, duplicate screening, calibration training, and consensus procedures were implemented to minimize selection errors, consistent with PRISMA 2020 recommendations.

**Eligibility criteria**

We included observational studies (cross-sectional, case-control, and cohort studies), experimental studies (including clinical trials), ecological studies, and case reports or case series that evaluated individuals of any age diagnosed with one or more WHO-listed NTDs and reported long-term sequelae or disabilities attributable to these conditions. Studies from all geographic regions were eligible for inclusion. The primary outcome of interest was the presence of permanent or long-term disability detected or reported after NTD diagnosis, whether directly resulting from the disease or from its secondary consequences. Studies were excluded if they did not include individuals affected by NTDs, did not report any form of disability or sequelae, involved non-human subjects, or were limited to reviews, editorials, letters, conference abstracts, posters, or grey literature. Articles for which full texts could not be obtained after attempts to contact the authors were also excluded.

**Definition of disability and sequelae**

For the purposes of this review, sequelae were defined as permanent anatomical or functional impairments resulting from NTDs. These included physical and motor disabilities; neurological impairments (including sensory, visual, auditory, or cognitive deficits); deformities of limbs or body segments; and chronic damage to organs or body systems that resulted in long-term functional limitations. This definition was intentionally broad to accommodate the heterogeneity of reporting across studies and to capture the multidimensional nature of disability associated with NTDs.

**Study selection**

The initial search strategy identified 958 records. After removing duplicates (n = 478), 480 records were screened independently by two reviewers (ADS and JSV) based on titles and abstracts. Disagreements were resolved by consensus, and when necessary, a third reviewer (CCLBB) was consulted. During the screening phase, 170 records were excluded. A total of 310 full-text articles were then assessed for eligibility by ADS and JSV. Of these, 180 were excluded for reasons including non-eligible publication types (reviews, editorials, letters, short reports, and comments), laboratory or in vitro studies, absence of NTDs, or lack of reported sequelae. Ultimately, 130 studies were included in the final analysis (Figure 1).

**[Insert Figure 1 here]**

**Data extraction**

Data extraction was independently performed by two reviewers using a standardized and piloted form. Extracted variables included study characteristics (author, year, country, income level, and design), population characteristics (sample size, age, sex, comorbidities, and co-infections), type of neglected tropical disease, disability outcomes, and number of individuals affected. Discrepancies were resolved by discussion and, when necessary, adjudication by a third reviewer. Duplicate extraction was implemented to minimize errors and improve data accuracy.

**Risk of bias and methodological quality assessment**

Given the heterogeneity of study designs, methodological quality was assessed using design-specific tools. Observational studies were evaluated using the Methodological Index for Non-Randomized Studies (MINORS) (19,20), while case reports and case series were assessed using the CARE checklist (20). Randomized clinical trials were evaluated using the Jadad scale (21). Rather than excluding studies based on quality scores, all eligible studies were retained to provide a comprehensive overview of the evidence. Methodological limitations were considered during interpretation of results. A summary of the risk of bias assessment is provided in the Supplementary Material.

**Data synthesis**

Due to substantial heterogeneity in study designs, populations, disability definitions, and outcome measures, a meta-analysis was not performed. Results were synthesized descriptively, focusing on the prevalence and types of disabilities reported across NTDs, as well as their distribution by disease, system affected, and geographic region.

**RESULTS**

**Study selection and characteristics of included studies**

The initial search strategy identified 958 records. After removing duplicates (n = 345) and applying the eligibility criteria, 130 studies were included in the final analysis (Figure 1). These studies encompassed a wide range of methodological designs, including case reports, case series, cross-sectional studies, case-control studies, cohort studies, clinical trials, ecological analyses, and retrospective studies. Collectively, the included studies covered 25 countries. Brazil contributed the largest number of publications (n = 73), accounting for more than half of all included studies. Other countries with multiple contributions included Sri Lanka and Colombia (approximately five studies each), followed by India (approximately four studies). The United Kingdom and the United States contributed a smaller number of studies (approximately three each).

This systematic review synthesized a substantial body of evidence, including 130 studies comprising 551,574 individuals across 25 countries. Overall, 29% of assessed individuals presented at least one disability, highlighting disability as a meaningful and under-recognized outcome across neglected tropical diseases. This distribution reflects both the global scope of NTDs and the regional concentration of specific conditions, such as leprosy and Chagas disease in Latin America, or trachoma and onchocerciasis in Africa. The characteristics of the included studies are summarized in Table 1.

**[Insert Table 1 here]**

The country with the highest number of studies of leprosy was Brazil, with a total of 60 studies, followed by snakebites (n=10) and Chagas Disease (n=8). However, the incidence of other NTDs was unevenly distributed, with wide distribution in countries in Africa, Asia and the Americas, and low or absent incidence in Europe and Oceania, respectively **(Figure 2)**.

**[Insert Figure 2 here]**

**Demographic profile of study populations**

The included studies encompassed a total of 551,574 individuals. Sample sizes ranged from single patient case reports to large-scale ecological studies with more than 390,000 participants. The proportion of female participants varied widely, from 12.5% to 100%, and mean age ranged from 6.2 years in a schistosomiasis trial to 68.9 years in a cross-sectional leprosy study conducted in Brazil. Despite this diversity, demographic data such as age and sex were frequently absent, particularly in older publications, limiting the possibility of conducting detailed subgroup analyses by sex or age group.

**Reported comorbidities**

Comorbidities were infrequently and inconsistently reported across studies. Among the reported conditions, respiratory diseases were the most frequent (0.2%, n = 1,019), followed by heart diseases (0.1%, n = 540). Mental health conditions, including anxiety and depression, were reported in 0.05% (n = 295) of individuals. Other comorbidities included hypertension (0.03%, n = 180), diabetes (0.02%, n = 149), and obesity (0.01%, n = 87). Despite the low reported frequencies, these findings likely reflect substantial underreporting across studies.

**[Insert Table 2 here]**

**Prevalence and types of disabilities**

The synthesis of the included studies revealed that long-term disabilities are a major consequence of NTDs. Overall, 29% (n = 158,104) of all evaluated individuals presented with at least one form of disability.

A wide range of additional sequelae were reported with lower prevalence. These included fever (0.24%, n = 1,319), appetite changes (0.19%, n = 1,034), abdominal pain (0.18%, n = 985), nerve injuries (0.17%, n = 964), motor dysfunctions (0.15%, n = 803), and fatigue (0.12%, n = 667). Respiratory and cardiovascular-related symptoms included dyspnea (0.09%, n = 437), cough (0.07%, n = 373), and arrhythmias or tachycardia (0.05%, n = 273). Physical and motor disability predominated (24.6%, n = 135,683), followed by visual disability (1.81%, n = 10,007). Cardiovascular, neurological, and psychosocial sequelae were also reported, broadening the spectrum of disability outcomes associated with neglected tropical diseases, followed by skin-related conditions (0.33%, n = 1,816) and gastrointestinal impairments (0.25%, n = 1,379).

Other sequelae encompassed a broad spectrum of manifestations. Neurological outcomes included nerve injuries (0.17%, n = 964), paresthesia (0.10%, n = 538), and cognitive or memory deficits (<0.01%). Psychological and social impairments were also present, such as depression (0.03%, n = 158), psychological disorders (0.02%, n = 135), and family or work-related emotional difficulties (0.01–0.02%). Functional and systemic symptoms were frequently observed, including gastrointestinal complaints (0.25%, n = 1,379), fever (0.24%, n = 1,319), and muscle pain (0.06%, n = 304).

**Disease-specific distribution of sequelae**

The distribution of disabilities varied considerably by disease (Figure 3). Leprosy accounted for most physical and motor impairments, consistent with its well-documented impact on the peripheral nervous system. Trachoma was the leading cause of visual impairment, followed by onchocerciasis. Cardiovascular impairments were overwhelmingly associated with Chagas disease, where chronic cardiomyopathy remains the hallmark condition. Snakebite envenoming was frequently reported as a cause of local tissue damage and persistent motor dysfunction, although prevalence estimates varied substantially across studies. Neurological sequelae were prominent in diseases such as neurocysticercosis and human African trypanosomiasis, manifesting as seizures, cognitive decline, or other forms of neutralizability.

**[Insert Figure 3 here]**

**Regional distribution of sequelae**

Regional patterns were also evident. In Latin America, leprosy and Chagas disease dominated the profile of disabilities, while in Africa, trachoma and onchocerciasis accounted for most of the reported impairments. In Asia, studies more frequently reported snakebite-related disabilities, as well as complications from leprosy and dengue. However, some WHO-listed NTDs, such as yaws, dracunculiasis, and scabies, were rarely, or not represented, in the included studies, highlighting persistent gaps in evidence in this manner.

**Risk of bias and reporting limitations**

The methodological quality of the included studies was highly heterogeneous. In general, more recent publications showed greater adherence to reporting standards, while older studies frequently failed to provide essential demographic information such as age and sex. Using the MINORS tool (19,20), most cross-sectional and cohort studies scored between 18 and 24 out of a maximum of 24 points, reflecting some incomplete reporting of endpoints, sample representativeness, and follow-up details. Case reports and series, assessed through the Critical Appraisal Checklist (20), often lacked clear descriptions of clinical pathways and standardized definitions of disability. The clinical trials assessed using the Jadad scale (21), demonstrated adequate methodological quality. A detailed summary of the risk of bias assessment is provided in **Supplementary Material 1**.

**DISCUSSION**

This systematic review provides a comprehensive overview of the disabilities associated with NTDs, synthesizing data from 130 studies and encompassing over half a million individuals. The findings reveal that physical and motor disabilities are the most frequently reported sequelae, affecting a substantial portion of the studied population. This underscores the lasting impact of NTDs on the functional capacity and quality of life of affected individuals. The high prevalence of these disabilities highlights a critical gap in the management of NTDs, which has historically focused more on incidence, mortality and transmission control than on long-term morbidity and rehabilitation (152). This review synthesizes evidence from a large and heterogeneous body of literature and reinforces that disability represents a substantial and under-recognized outcome across neglected tropical diseases. The descriptive estimate that 29% of individuals presented at least one disability highlights the magnitude of long-term functional consequences beyond infection control.

The predominance of physical and motor disabilities (24.6%) in our findings aligns with the well-documented impact of diseases like leprosy, which was the most frequently addressed NTD in the included studies. Leprosy is known to cause significant nerve damage, leading to sensory loss, muscle weakness, and visible deformities, which severely impair motor function (153–155). Our review included a large number of studies from Brazil, a country with a high burden of leprosy, which may have contributed to the high prevalence of physical disabilities observed. A large cohort study in Brazil, for instance, found that 27% of new leprosy cases presented with some degree of physical disability (Grades 1 or 2), with multibacillary forms of the disease being associated with an over 8-fold increased risk of Grade 2 disability (152,156). This emphasizes that beyond the infectious process itself, the resulting chronic disabilities constitute a major public health challenge, perpetuating cycles of poverty and social stigma (152).

Visual impairment was the second most common disability identified in this review (1.81%). This is largely attributable to diseases like trachoma and onchocerciasis. Trachoma, caused by Chlamydia trachomatis, is the world's leading infectious cause of blindness. Repeated infections lead to trachomatous trichiasis (TT), where the eyelashes turn inward and scrape the cornea, eventually causing corneal opacity (CO) and irreversible blindness (157). A recent meta-analysis estimated that, globally, over 1.9 million people are blind or have severe to moderate vision impairment due to trachoma (157). Although the prevalence of trachomatous blindness has been decreasing due to successful control strategies, it remains a significant problem in many endemic areas, particularly in Africa (157). The impact of visual impairment extends beyond the loss of sight, affecting individuals' ability to work, their social participation, and their overall quality of life.

Cardiovascular complications, although reported less frequently in the included studies, are a hallmark of Chagas disease, another of the most studied NTDs in this review. Chronic Chagas cardiomyopathy is a severe and often fatal consequence of infection with Trypanosoma cruzi, affecting up to 30% of chronically infected individuals (158). It leads to a range of cardiovascular issues, including heart failure, arrhythmias, stroke, and sudden death. The pathogenesis is complex, involving a parasite-driven immune response, direct tissue damage, and microvascular derangements that result in progressive myocardial fibrosis and dysfunction (158). The burden of chagas disease is substantial, not only in Latin America but also in non-endemic countries due to migration (158). The disabilities resulting from cardiac complications, such as fatigue, dyspnea, and reduced functional capacity, severely limit the daily activities of patients and impose a significant economic burden on health systems.

This review also identified a wide range of other sequelae, including neurological, psychological, and social impacts, which are often under-recognized. Neurological impairments, for example, are not limited to the peripheral nerve damage in leprosy but are also a feature of diseases like neurocysticercosis and human African trypanosomiasis (152,159). Furthermore, the social stigma and mental health consequences associated with the disfiguring and disabling nature of many NTDs are profound (152,159). Depression, anxiety, and social exclusion are common among people affected by NTDs, further exacerbating their suffering and hindering their access to care and participation in society (155).

The methodological heterogeneity observed in the studies included in this review poses a significant challenge to evidence synthesis and policymaking. The lack of standardized disability definitions and the variety of assessment tools used make it difficult to compare data across different studies and regions, likely resulting in an underestimation of the true burden of disability. In response to this gap, efforts have been made to develop and validate cross-NTD morbidity and disability assessment toolkits, such as the "cross-NTD toolkit" (160).

Despite the substantial burden of long-term disabilities identified in this review, access to rehabilitation services for individuals affected by NTDs remains profoundly limited and inadequately integrated into health systems. In most endemic settings, care models continue to prioritize acute disease control, case detection, and pharmacological treatment, while long-term functional outcomes and rehabilitation needs receive minimal clinical attention (161). Consequently, chronic sequelae, such as motor impairment, sensory loss, visual deficits, cognitive limitations, and psychosocial consequences, are frequently under-recognized or insufficiently addressed by physicians and healthcare teams managing NTDs. This gap often results in delayed diagnosis of disability, low referral rates to rehabilitation services, and fragmented or absent continuity of care. These shortcomings directly contradict the principles of the WHO Rehabilitation 2030 initiative and Universal Health Coverage, which emphasize rehabilitation as an essential health service across the life course. Furthermore, the scarcity of rehabilitation professionals, assistive technologies, and community-based rehabilitation programs, particularly in low- and middle-income countries where NTDs have higher incidence, exacerbates structural inequities in access. Financial barriers, geographic isolation, limited workforce capacity, and insufficient policy prioritization further restrict service availability. Addressing these gaps is essential to reduce avoidable disability, improve functional independence, and ensure that NTD control strategies move beyond infection elimination toward truly patient-centered and disability-inclusive care.

This systematic review has several limitations. First, the included studies were highly heterogeneous in terms of design, methodology, disability definitions, and assessment instruments, which limited comparability and precluded meta-analysis. Second, there was substantial underreporting of disabilities, and definitions varied widely across studies, hampering comparison of findings. The frequent use of “not reported” (NR) for key demographic data, such as age and sex, particularly in older studies, also limited subgroup analyses. In addition, the evidence base was dominated by leprosy and by studies from a limited number of countries, particularly Brazil, which may restrict generalizability to other neglected tropical diseases and settings. Many included studies were observational and facility-based, raising the possibility of selection bias toward more severe cases, as individuals with milder or asymptomatic conditions are less likely to seek care or be captured in clinical settings. Finally, limited longitudinal data, underreporting of psychosocial outcomes, and potential publication bias may have influenced the overall estimates.

**CONCLUSIONS**

The findings of this systematic review demonstrate that disabilities are a major and often neglected consequence of NTDs. The high prevalence of physical, visual, and other impairments highlights the urgent need to integrate rehabilitation and disability-inclusive development into NTD control programs. A paradigm shift is needed, moving beyond a purely biomedical approach to a more holistic one that addresses the long-term physical, psychological, and social needs of people affected by NTDs, alongside with reintegration into daily-life, social and work-related activities. Future research should focus on developing and evaluating effective and scalable rehabilitation interventions, as well as on improving the standardization of disability assessment and reporting in NTD studies.

**REFERENCES**

1.	Neglected tropical diseases [Internet]. [citado 4 de junho de 2025]. Disponível em: https://www.who.int/news-room/questions-and-answers/item/neglected-tropical-diseases

2.	CA J, P VBK, Kandi V, N G, K S, Dharshini D, et al. Neglected Tropical Diseases: A Comprehensive Review. Cureus. 10 de fevereiro de 2024;16(2):e53933. doi:10.7759/CUREUS.53933 PubMed PMID: 38468991.

3.	Rodrigues A, Santos-Gomes G. Editorial: Neglected tropical diseases: tackling the challenges of a global world. Frontiers in Toxicology. 2024;6:1417438. doi:10.3389/FTOX.2024.1417438 PubMed PMID: 38745693.

4.	Ogieuhi IJ, Ajekiigbe VO, Aremu SO, Okpujie V, Bassey PU, Babalola AE, et al. Global partnerships in combating tropical diseases: assessing the impact of a U.S. withdrawal from the WHO. Trop Med Health. 1o de dezembro de 2025;53(1):36. doi:10.1186/S41182-025-00722-8 PubMed PMID: 40065473.

5.	Ending NTDs together towards 2030 [Internet]. [citado 4 de junho de 2025]. Disponível em: https://www.who.int/teams/control-of-neglected-tropical-diseases/ending-ntds-together-towards-2030

6.	Álvarez-Hernández DA, Rivero-Zambrano L, Martínez-Juárez LA, García-Rodríguez-Arana R. Overcoming the global burden of neglected tropical diseases. Ther Adv Infect Dis. 2020;7:2049936120966449. doi:10.1177/2049936120966449 PubMed PMID: 33178435.

7.	CA J, P VBK, Kandi V, N G, K S, Dharshini D, et al. Neglected Tropical Diseases: A Comprehensive Review. Cureus. 10 de fevereiro de 2024;16(2):e53933. doi:10.7759/CUREUS.53933 PubMed PMID: 38468991.

8.	Chen J, Tian X, Guo D, Gu H, Duan Y, Li D. Global trends and burdens of neglected tropical diseases and malaria from 1990 to 2021: a systematic analysis of the Global Burden of Disease Study 2021. BMC Public Health. 1o de dezembro de 2025;25(1):1307. doi:10.1186/S12889-025-22477-X PubMed PMID: 40197173.

9.	Winkler AS, Klohe K, Schmidt V, Haavardsson I, Abraham A, Prodjinotho UF, et al. Neglected tropical diseases – the present and the future. Tidsskr Nor Laegeforen. 2018;138(3):10.4045/tidsskr.17.0678. doi:10.4045/TIDSSKR.17.0678 PubMed PMID: 29411594.

10.	Hunter P. Tropical diseases and the poor: Neglected tropical diseases are a public health problem for developing and developed countries alike. EMBO Rep. 2014;15(4):347. doi:10.1002/EMBR.201438652 PubMed PMID: 24652851.

11.	Bharadwaj M, Bengtson M, Golverdingen M, Waling L, Dekker C. Diagnosing point-of-care diagnostics for neglected tropical diseases. PLoS Negl Trop Dis. 1o de junho de 2021;15(6):e0009405. doi:10.1371/JOURNAL.PNTD.0009405 PubMed PMID: 34138846.

12.	Kuper H. Neglected tropical diseases and disability—what is the link? Trans R Soc Trop Med Hyg. 1o de dezembro de 2019;113(12):839. doi:10.1093/TRSTMH/TRZ001 PubMed PMID: 30892653.

13.	Weiss MG. Stigma and the Social Burden of Neglected Tropical Diseases. PLoS Negl Trop Dis. maio de 2008;2(5):e237. doi:10.1371/JOURNAL.PNTD.0000237 PubMed PMID: 18478049.

14.	Koschorke M, Al-Haboubi YH, Tseng PC, Semrau M, Eaton J. Mental health, stigma, and neglected tropical diseases: A review and systematic mapping of the evidence. Frontiers in Tropical Diseases. 11 de outubro de 2022;3:808955. doi:10.3389/FITD.2022.808955/XML/NLM

15.	van Wijk R, van Selm L, Barbosa MC, van Brakel WH, Waltz M, Philipp Puchner K. Psychosocial burden of neglected tropical diseases in eastern Colombia: an explorative qualitative study in persons affected by leprosy, cutaneous leishmaniasis and Chagas disease. Global Mental Health. 2021;8:e21. doi:10.1017/GMH.2021.18 PubMed PMID: 34249368.

16.	Alderton DL, Ackley C, Trueba ML. The psychosocial impacts of skin-neglected tropical diseases (SNTDs) as perceived by the affected persons: A systematic review. PLoS Negl Trop Dis. 1o de agosto de 2024;18(8):e0012391. doi:10.1371/JOURNAL.PNTD.0012391 PubMed PMID: 39093848.

17.	Hofstraat K, Van Brakel WH. Social stigma towards neglected tropical diseases: A systematic review. Int Health. 1o de dezembro de 2015;8:i53–70. doi:10.1093/INTHEALTH/IHV071, PubMed PMID: 26940310.

18.	Page MJ, McKenzie JE, Bossuyt PM, Boutron I, Hoffmann TC, Mulrow CD, et al. The PRISMA 2020 statement: an updated guideline for reporting systematic reviews. BMJ. 29 de março de 2021;372. doi:10.1136/BMJ.N71 PubMed PMID: 33782057.

19.	Slim K, Nini E, Forestier D, Kwiatkowski F, Panis Y, Chipponi J. Methodological index for non‐randomized studies ( MINORS ): development and validation of a new instrument. ANZ J Surg. 8 de setembro de 2003;73(9):712–6. doi:10.1046/j.1445-2197.2003.02748.x

20.	Gagnier JJ, Kienle G, Altman DG, Moher D, Sox H, Riley D. The CARE Guidelines: Consensus-based Clinical Case Reporting Guideline Development. Glob Adv Health Med. 1o de setembro de 2013;2(5):38–43. doi:10.7453/gahmj.2013.008

21.	Jadad AR, Moore RA, Carroll D, Jenkinson C, Reynolds DJM, Gavaghan DJ, et al. Assessing the quality of reports of randomized clinical trials: Is blinding necessary? Control Clin Trials. fevereiro de 1996;17(1):1–12. doi:10.1016/0197-2456(95)00134-4

22.	Pires KL, Hammerle MB, Lima RB, Hammerle MB, Caetano MR, Araújo LF, et al. Polineuropatia desmielinizante como manifestação inicial isolada de hanseníase. Medicina (Ribeirão Preto). 27 de abril de 2020;53(1):73–9. doi:10.11606/issn.2176-7262.v53i1p73-79

23.	Parente Y de DM, Castro AL de, Araújo FB de, Teixeira AC, Lima ÍC, Daher EDF. Acute renal failure by rapidly progressive glomerulonephritis with IgA deposition in a patient concomitantly diagnosed with multibacillary Hansen’s disease: a case report. Brazilian Journal of Nephrology. março de 2019;41(1):152–6. doi:10.1590/2175-8239-jbn-2018-0056

24.	Medina Ortega A, López Valencia D, Vásquez Arteaga LR. Recurrent neurocysticercosis of the frontal lobe. Case report. Case reports. 1o de janeiro de 2018;4(1):46–53. doi:10.15446/cr.v4n1.65860

25.	Díaz Ramírez GS, Morales Plaza CD, Matta Cortés L. Glomerulonefritis por inmunocomplejos en un paciente con lepra. Infectio. 20 de janeiro de 2017;21(1). doi:10.22354/in.v21i1.641

26.	Dametto E, Zuffo S, Juhas M, Malic MK. NEUROCYSTICERCOSIS IN ASPLENIC PATIENT, CASE REPORT. Revista Brasileira de Neurologia. 2016;52:36–9.

27.	Hernández-Beltrán N, Rodríguez Quintana J, Mantilla Sylvain F. Síndrome de Guillain-Barré en un paciente con enfermedad de Chagas aguda. Infectio. outubro de 2015;19(4):172–4. doi:10.1016/j.infect.2015.01.001

28.	Seijo A, Luppo V, Morales A, Gancedo E, Romer Y, Correa J, et al. TENOSINOVITIS POR VIRUS CHIKUNGUNYA. Medicina (Buenos Aires). 2014;74:476–8.

29.	Rigo R, Rigo L, Honer M. Change in renal function and pancreatitis: adverse effects of the treatment of visceral leishmaniasis. Arquivos de Ciências da Saúde. 2008;15(3):139–41.

30.	Andrade Filho AS, Queiroz AC, Freire ACC, Lima LCS, S. Filho CA, Amado IN, et al. Pseudotumoral form of neuroschistosomiasis: report of three cases. Brazilian Journal of Infectious Diseases. agosto de 2007;11(4):435–8. doi:10.1590/S1413-86702007000400014

31.	Parajuli N, Adhikary S, Karki A, Tiwari S. Case Report: Erysipeloid Cutaneous Leishmaniasis Treated with Oral Miltefosine. Am J Trop Med Hyg. 3 de fevereiro de 2021;104(2):643–5. doi:10.4269/ajtmh.20-0918

32.	Pradhan A, Tobgay T, Dorjee S, Wangdi T, Zhou G, Karunaweera ND. Atypical Presentation of Post-Kala-Azar Dermal Leishmaniasis in Bhutan. Case Rep Dermatol Med. 21 de agosto de 2020;2020:1–4. doi:10.1155/2020/8899586

33.	Pallangyo P, Bhalia S, Simelane NN, Lyimo F, Swai HJ, Mkojera ZS, et al. Massive Bilateral Hydroureteronephrosis and End-Stage Renal Disease Ensuing From Chronic Schistosomiasis: A Case Report. J Investig Med High Impact Case Rep. 5 de janeiro de 2020;8. doi:10.1177/2324709620910912

34.	Dalugama C, Gawarammana IB. Lessons learnt from managing a case of dengue hemorrhagic fever complicated with acute liver failure and acute kidney injury: a case report. J Med Case Rep. 8 de dezembro de 2018;12(1):215. doi:10.1186/s13256-018-1766-0

35.	Sadiq N, Naqaish T, Arif A, Mohammad K, Jalis M. CENTRAL RETINAL ARTERY OCCLUSION SECONDARY TO DENGUE FEVER. Journal of Ayub Medical College Abbottabad. 2014;26.

36.	Chang J, Cheng C, Asok K, Fong K, Chee S, Tan C. Visual disturbances in dengue fever: an answer at last? Singapore Med J. 2007.

37.	Tan SY, Kumar G, Surrun SK, Ong YY. Dengue maculopathy: A case report. Travel Med Infect Dis. janeiro de 2007;5(1):62–3. doi:10.1016/j.tmaid.2006.01.017

38.	Bentes KO, de Amorim RLO, Barbosa FBA, Ratis da Silva VCP, Valente J, Almeida-Val F, et al. Long-term disability after cerebral ischemic stroke following a Bothrops atrox snakebite in the Brazilian Amazon. Toxicon. agosto de 2024;247:107793. doi:10.1016/j.toxicon.2024.107793

39.	Joshy S, Chaitra MC. Ocular sequelae following snakebite envenomation. The Pan-American Journal of Ophthalmology. setembro de 2024;6(3). doi:10.4103/pajo.pajo_71_24

40.	Rhee TK, Han J Il. Use of Optical Coherence Tomography to Evaluate Visual Acuity and Visual Field Changes in Dengue Fever. Korean Journal of Ophthalmology. 2014;28(1):96. doi:10.3341/kjo.2014.28.1.96

41.	Koh YT, Sanjay S. Characteristics and Ophthalmic Manifestations of the Classic Dengue Fever Epidemic in Singapore (2005–2006). Asia-Pacific Journal of Ophthalmology. 2013;2(2):99–103. doi:10.1097/APO.0b013e31828a1917

42.	Espiritu CG, Gelber R, Ostler HB. Chronic anterior uveitis in leprosy: an insidious cause of blindness. British Journal of Ophthalmology. 1o de maio de 1991;75(5):273–5. doi:10.1136/bjo.75.5.273

43.	Pazin-Filho A, Romano MMD, Almeida-Filho OC, Furuta MS, Viviani LF, Schmidt A, et al. Minor segmental wall motion abnormalities detected in patients with Chagas’ disease have adverse prognostic implications. Brazilian Journal of Medical and Biological Research. abril de 2006;39(4):483–7. doi:10.1590/S0100-879X2006000400008

44.	Dantas RO, Aprile LRO, Aben-Athar CG, Miranda ALM. Esophageal striated muscle contractions in patients with Chagas’ disease and idiopathic achalasia. Brazilian Journal of Medical and Biological Research. junho de 2002;35(6):677–83. doi:10.1590/S0100-879X2002000600007

45.	Mangone CA, Sica REP, Pereyra S, Genovese O, Segura E, Riarte A, et al. Cognitive impairment in human chronic Chagas’ disease. Arq Neuropsiquiatr. junho de 1994;52(2):200–3. doi:10.1590/S0004-282X1994000200008

46.	Zicker F. Chagas’ disease and social security: a case-control study in an urban area, Goiás, Brazil. Rev Saude Publica. agosto de 1988;22(4):281–7. doi:10.1590/S0034-89101988000400004

47.	Barda B, Coulibaly JT, Hatz C, Keiser J. Ultrasonographic evaluation of urinary tract morbidity in school-aged and preschool-aged children infected with Schistosoma haematobium and its evolution after praziquantel treatment: A randomized controlled trial. PLoS Negl Trop Dis. 21 de fevereiro de 2017;11(2):e0005400. doi:10.1371/journal.pntd.0005400

48.	Masud H, Qureshi TQ, Dukley M. Effects of Ivermectin with and without Doxycycline on Clinical Symptoms of Onchocerciasis. Journal of the College of Physicians and Surgeons. 2009;19:34–8.

49.	Wijesinghe CA, Williams SS, Kasturiratne A, Dolawaththa N, Wimalaratne P, Wijewickrema B, et al. A Randomized Controlled Trial of a Brief Intervention for Delayed Psychological Effects in Snakebite Victims. PLoS Negl Trop Dis. 11 de agosto de 2015;9(8):e0003989. doi:10.1371/journal.pntd.0003989

50.	Melese M. Characteristics of trichiasis patients presenting for surgery in rural Ethiopia. British Journal of Ophthalmology. 1o de setembro de 2005;89(9):1084–8. doi:10.1136/bjo.2005.066076

51.	Agbenorku P. Multicenter Study of Buruli Ulcer Disabilities in the Head and Neck Region. Plast Surg Int. 26 de abril de 2011;2011:1–5. doi:10.1155/2011/647418

52.	de-Araujo BHJM, Hazime PBN, Galeno FJV, Candeira LN, Sampaio MF, Hazime FA. Clinical manifestations in patients with musculoskeletal pain post-chikungunya. Brazilian Journal Of Pain. 2019;2(4). doi:10.5935/2595-0118.20190060

53.	Ribeiro G de C, Lana FCF. INCAPACIDADES FÍSICAS EM HANSENÍASE: CARACTERIZAÇÃO, FATORES RELACIONADOS E EVOLUÇÃO. Revista Cogitare Enfermagem. 2015;20:496–503.

54.	Borges-Pereira J, Xavier SS, Pirmez C, Coura JR. Doença de Chagas em Virgem da Lapa, Minas Gerais, Brasil. IV. Aspectos clínicos e epidemiológicos do aneurisma ventricular esquerdo. Rev Soc Bras Med Trop. outubro de 1998;31(5):457–63. doi:10.1590/S0037-86821998000500006

55.	Bowman R. Natural history of trachomatous scarring in the Gambia Results of a 12-year longitudinal follow-up. Ophthalmology. dezembro de 2001;108(12):2219–24. doi:10.1016/S0161-6420(01)00645-5

56.	Bernardes CA, Santos AF, Padovani CTJ, Santos LF, Filho GH. Incapacidade Física em Hansenianos de Campo Grande - Mato Grosso do Sul. Hansenologia Internationalis . 2009;34(1):17–25.

57.	Klis S, Stienstra Y, Phillips RO, Abass KM, Tuah W, van der Werf TS. Long Term Streptomycin Toxicity in the Treatment of Buruli Ulcer: Follow-up of Participants in the BURULICO Drug Trial. PLoS Negl Trop Dis. 13 de março de 2014;8(3):e2739. doi:10.1371/journal.pntd.0002739

58.	Costa RMPG, Fernandes MA, Zagonel IPS. Transitions experienced by people living with limitations resulting from leprosy: a research-care study. Rev Bras Enferm. 2024;77(5). doi:10.1590/0034-7167-2023-0229

59.	Bomtempo CF, Ferrari SMF, Grossi MA de F, Lyon S. Evolução do grau de incapacidade física e do escore olhos, mãos e pés em casos novos de hanseníase. Hansenologia Internationalis: hanseníase e outras doenças infecciosas. 19 de setembro de 2023;48:1–17. doi:10.47878/hi.2023.v48.37331

60.	Machado GLR, Castro RQ, Forechi L, Souza H da C, Fonseca DS, Garcia MAC. The impact of Chikungunya chronic arthralgia on women’s upper limbs motor function: a cross-sectional study. Fisioterapia e Pesquisa. outubro de 2022;29(4):412–20. doi:10.1590/1809-2950/22011229042022en

61.	Chaves AF de CP, Araújo OD de, Sousa EC da CL, Cardoso JA, Rodrigues É de A, Moura EH. Magnitude dos episódios reacionais na hanseníase em serviço ambulatorial especializado. Hansenologia Internationalis: hanseníase e outras doenças infecciosas. 21 de novembro de 2022;47:1–12. doi:10.47878/hi.2022.v47.37318

62.	Matos TS, Moura JC de, Fernandes TRM de O, Souza CDF de. Epidemiological, neurofunctional profile and prevalence of factors associated with the occurrence of physical disabilities due to leprosy in a reference center in Northeast Brasil: a sectional study. Rev Assoc Med Bras. janeiro de 2021;67(1):19–25. doi:10.1590/1806-9282.67.01.20200139

63.	D’Azevedo SSP, Santos DCM dos, Arruda GA de, Barbosa JC, Alves MGT, Souza NMN. Profile of functionality of persons affected by leprosy. Rev Rene. 12 de julho de 2021;22:e61702. doi:10.15253/2175-6783.20212261702

64.	Campos FA, Magalhães ML, Moreira HT, Pavão RB, Lima MO, Lago IM, et al. Estudo Comparativo da Doença Coronariana Microvascular Causada por Doença de Chagas e por Outras Etiologias. Arq Bras Cardiol. 14 de dezembro de 2020;115(6):1094–101. doi:10.36660/abc.20200381

65.	Sobral LL, Santos MC de S, Rocha LS de O, Callegari B, Souza G da S, Teodori RM. Maximum voluntary muscle contraction and fatigue in multibacillary leprosy. Fisioterapia em Movimento. 2020;33. doi:10.1590/1980-5918.33.ao08

66.	Abella J, Rojas Á, Rojas C, Rondón F, Medina Y, Peña M, et al. Clinical and immunological features of post-chikungunya virus chronic arthritis and its effect on functional ability and quality of life in a cohort of Colombian patients. Revista Colombiana de Reumatología (English Edition). outubro de 2019;26(4):253–9. doi:10.1016/j.rcreue.2019.12.001

67.	Silva Rezende da Silva J, Proença Palmeira I, Margareth Moita Sá A, Vidal Nogueira LM, Rodrigues Ferreira AM. Variáveis clínicas associadas ao grau de incapacidade física na hanseníase. Revista Cuidarte. 20 de dezembro de 2018;10(1). doi:10.15649/cuidarte.v10i1.618

68.	Robinet MET, Rodríguez HM, Pupo NL, Tablada DM, González OP. Aspectos clínicos y epidemiológicos en pacientes timorenses con enfermedad de Hansen. Revista MEDISAN. 2019;23(1).

69.	Silva Rezende da Silva J, Proença Palmeira I, Moita Sá AM, Vidal Nogueira LM, Rodrigues Ferreira AM. Fatores sociodemográficos associados ao grau de incapacidade física na hanseníase. Revista Cuidarte. 5 de setembro de 2018;9(3):2338–48. doi:10.15649/cuidarte.v9i3.548

70.	Loiola HADB, Aquino DMC de, Cardoso LSP, Paiva M de FL, Coutinho NPS, Dias RS. Perfil epidemiológico, clínico e qualidade de vida de crianças com hanseníase em um município hiperendêmico. Revista Enfermagem UERJ. 5 de outubro de 2018;26:e32251. doi:10.12957/reuerj.2018.32251

71.	Silva DDB da, Tavares CM, Gomes NMC, Cardoso AC, Arcêncio RA, Nogueira PSF. Leprosy in the elderly population of Alagoas. Revista Brasileira de Geriatria e Gerontologia. outubro de 2018;21(5):553–61. doi:10.1590/1981-22562018021.180076

72.	Aben-Athar CYUP, Lima SS, Ishak R, Vallinoto ACR. Assessment of the sensory and physical limitations imposed by leprosy in a Brazilian Amazon Population. Rev Soc Bras Med Trop. março de 2017;50(2):223–8. doi:10.1590/0037-8682-0493-2016

73.	Basso ME de M, Silva RLF. Perfil clínico-epidemiológico de pacientes acometidos pela hanseníase atendidos em uma unidade de referência. Revista da Sociedade Brasileira de Clínica Médica. janeiro de 2017;15(1):27–32.

74.	Belkiman-Pedro MF, Marciano LHSC, Marques T, Quaggio CM da P, Nardi SMT. DESEMPENHO FUNCIONAL DE PACIENTES COM DEFORMIDADES VISÍVEIS NA HANSENÍASE. Hansenologia Internationalis: . 2017;42:19–27.

75.	Arco R Del, Nardi SMT, Bassi TG, Paschoal VDA. Diagnosis and medical treatment of neuropathic pain in leprosy. Rev Lat Am Enfermagem. 2016;24(0). doi:10.1590/1518-8345.0676.2731

76.	Gaudenci EM, Nardelli GG, Neto OP de A, Malaquias BSS, Carvalho BT, Pedrosa LAK. QUALIDADE DE VIDA, SINTOMAS DEPRESSIVOS E INCAPACIDADE FÍSICA DE PACIENTES COM HANSENÍASE. Hansenol Int. 2015;40(2):48–58.

77.	Monteiro LD, Alencar CH, Barbosa JC, Novaes CCBS, Silva R de CP da, Heukelbach J. Limited activity and social participation after hospital discharge from leprosy treatment in a hyperendemic area in north Brazil. Revista Brasileira de Epidemiologia. março de 2014;17(1):91–104. doi:10.1590/1415-790X201400010008ENG

78.	Pelarigo JGT, Prado RBR, Nardi SMT, Quaggio CM da P, Camargo LHS, Marciano LHSC. DECLÍNIO COGNITIVO, INDEPENDÊNCIA FUNCIONAL E SINTOMAS DEPRESSIVOS EM IDOSOS COM HANSENÍASE. Hansenol Int. 2014;39:30–9.

79.	Carvalho T de J. FOLDER DE EXERCÍCIOS TERAPÊUTICOS PARA PESSOAS COM INCAPACIDADES FÍSICAS CAUSADAS POR HANSENÍASE. Hansenologia Internationalis . 2014;39:3–18.

80.	Araújo MM, Silva JH de S, Gomes ACS, Lopes LRS, Marques RB. PERFIL CLÍNICO-EPIDEMIOLÓGICO DE PACIENTES QUE ABANDONARAM O TRATAMENTO DE HANSENÍASE. Hansenologia Internationalis . 2014;39:55–63.

81.	Monteiro LD, Alencar CHM de, Barbosa JC, Braga KP, Castro MD de, Heukelbach J. Incapacidades físicas em pessoas acometidas pela hanseníase no período pós-alta da poliquimioterapia em um município no Norte do Brasil. Cad Saude Publica. maio de 2013;29(5):909–20. doi:10.1590/S0102-311X2013000900009

82.	Carvalho MAJ, Lopes NTB, Santos TS dos, Santos K da S, Farnocchi PG, Tavares CM. AVALIAÇÃO DAS INCAPACIDADES FÍSICAS EM EX-PORTADORES DE HANSENÍASE DA ÉPOCA DO ISOLAMENTO COMPULSÓRIO. Hansenologia Internationalis . 2013;38:47–55.

83.	Baldan SS, Santos BM de O. HANSENÍASE: UMA ABORDAGEM NA PERSPECTIVA DE PROMOÇÃO DE SAÚDE. Hansenologia Internationalis . 2012;37:11–21.

84.	Pieri FM, Ramos ACV, Crispim J de A, Pitiá AC de A, Rodrigues LBB, Silveira TR dos S, et al. FATORES ASSOCIADOS ÀS INCAPACIDADES EM PACIENTES DIAGNOSTICADOS DE HANSENÍASE: UM ESTUDO TRANSVERSAL. Hansenologia Internationalis. 2012;37:22–30.

85.	Silva RS de O, Serra SMF da S, Gonçalves E da G do R, Silva AR da. HANSENÍASE NO MUNICÍPIO DE BURITICUPU, ESTADO DO MARANHÃO, BRASIL: ESTUDO DE INCAPACIDADES EM INDIVÍDUOS NO PÓS-ALTA. Hansenologia Internationalis. 2012;37(2):54–60.

86.	Guerrero MI, Muvdi S, León CI. Retraso en el diagnóstico de lepra como factor pronóstico de discapacidad en una cohorte de pacientes en Colombia, 2000 - 2010. Revista Panamericana de Salud Pública. fevereiro de 2013;33(2):137–43. doi:10.1590/S1020-49892013000200009

87.	Budel AR, Raymundo AR, Costa CF da, Gerhardt C, Pedri LE. Perfil dos pacientes acometidos pela hanseníase atendidos no Ambulatório de Dermatologia do Hospital Evangélico de Curitiba. An Bras Dermatol. outubro de 2011;86(5):942–6. doi:10.1590/S0365-05962011000500012

88.	Seijo A, Morales A, Poustis G, Romer Y, Efron E, Vilora G, et al. BROTE DE ENCEFALITIS DE SAN LUIS EN EL AREA METROPOLITANA BUENOS AIRES. Medicina (Buenos Aires). 2011;71.

89.	Raposo MT, Caminha AV de Q, Heukelbach J, Sánchez-González MÁ, Medeiros JLA de, Nemes MIB. Assessment of physical impairments in leprosy patients: a comparison between the world health organization (who) disability grade and the Eye-Hand-Foot Score. Rev Inst Med Trop Sao Paulo. abril de 2011;53(2):77–81. doi:10.1590/S0036-46652011000200004

90.	de Sousa NP, Silva MI de B, Lobo CG, Barboza MC de C, Abdon AP de V. Análise da qualidade de vida em pacientes com incapacidades funcionais decorrentes de hanseníase. Hansenologia Internationalis . 2011;36:11–6.

91.	Nardi SMT, Cruz LP da, Pedro H da SP, Marciano LHSC, Pachoal VD. Avaliação das deficiências físicas em pessoas com hanseníase empregando dois indicadores: grau de incapacidades e eyes-hands-feet. Hansenologia Internationalis . 2011;36:9–15.

92.	Rodini FCB, Gonçalves M, Barros AR de SB, Mazzer N, Elui VMC, Fonseca M de CR. Prevenção de incapacidade na hanseníase com apoio em um manual de autocuidado para pacientes. Fisioterapia e Pesquisa. junho de 2010;17(2):157–66. doi:10.1590/S1809-29502010000200012

93.	Silva CP, Del Carlo CH, Oliveira Junior MT de, Scipioni A, Strunz-Cassaro C, Ramirez JAF, et al. Por que os portadores de cardiomiopatia chagásica têm pior evolução que os não-chagásicos? Arq Bras Cardiol. dezembro de 2008;91(6). doi:10.1590/S0066-782X2008001800006

94.	Barbosa JC, Ramos Jr AN, Alencar M de JF, Castro CGJ de. Pós-alta em Hanseníase no Ceará: limitação da atividade funcional, consciência de risco e participação social. Rev Bras Enferm. novembro de 2008;61(spe):727–33. doi:10.1590/S0034-71672008000700012

95.	Cunha MHC da, Macedo GM de, Batista K de NM, Xavier MB, Neto S de S, Nascimento FS do N e. Avaliação Clínico-Epidemiológica em pacientes multibacilares em uma unidade de referência de hansenologia da Amazônia. Hansenologia Internationalis . 2008;33:9–16.

96.	Goldschmidt P, Zago VV, Vargas LD, Garcia LE, Montoya CM, Peralta B, et al. Chlamydia trachomatis in the conjunctiva of children living in three rural areas in Mexico. Revista Panamericana de Salud Publica. 2007;22:29–34.

97.	Escarabel CM, Alvarez RRA, Carvalho GA, Moreira D. ESTUDO COMPARATIvO DO ÍNDICE DE IN-CAPACIDADES EM PACIENTES TRATADOSCOM 24 E 12 DOSES DE POLIQUIMIOTERA-PIA PADRÃO – OMS, PACIENTES ATENDI-DOS NO PLANO PILOTO DE BRASÍLIA – DF. Hansenologia Internationalis . 2007;32:163–74.

98.	Carvalho G de A. Avaliação de incapacidades físicas neuro-músculo-esqueléticas em pacientes do Distrito Federal com hanseníase. Portal Regional da BVS. 1999;1.

99.	Maradei J, Santos PM dos, Santos RCR dos, Olivalves SMR, Abreu MT de. Complicações oculares como causa de incapacidade visual na hanseníase. Arquivo Brasileiro de Oftalmologia. 1998;61(1):4–11.

100.	Mol MM, Miedema JM, van Wijk R, Agarwal A, Nayak PK, Tiwari RK, et al. Impact of basic psychological support on stigma and the mental well-being of people with disabilities due to leprosy and lymphatic filariasis: a postintervention evaluation study. Int Health. 20 de dezembro de 2023;15(Supplement_3):iii70–8. doi:10.1093/inthealth/ihad100

101.	Vêncio D. Estudo do ofidismo em Goiás: comprometimento da funçäo renal . Rev Goiana Med. julho de 1988;34(4):95–116.

102.	Baranwal V, Shyamsundar K, Kabuyaya V, Biswas J, Vannadil H. Study of onchocerciasis-related visual impairment in North Kivu province of the Democratic Republic of Congo in Africa. Indian J Ophthalmol. 2020;68(5):890. doi:10.4103/ijo.IJO_1653_18

103.	Pedrazzani ES, Maluf SA, Pedroso M, Toyoda CY. Prevençäo de incapacidades em hanseníase: realidade numa unidade sanitária. Hanseniasis International. janeiro de 1985;10(1):10–22.

104.	Olamiju FO, Mogaji HO, Bjørn MT, Marcus AJ, Oduwa V, Olamiju OJ, et al. Preliminary assessment of Onchocerca-induced visual impairment using clinical fundus camera in Gashaka local government area of Taraba state, north eastern Nigeria. Parasite Epidemiol Control. maio de 2023;21:e00296. doi:10.1016/j.parepi.2023.e00296

105.	Getahun Abayo, Girum W. Gessesse, Tsedeke Asaminew. Prevalence and Pattern of Ocular Morbidity among School Children in Southern Ethiopia. Ethiop J Health Sci. 1o de julho de 2021;31(4). doi:10.4314/ejhs.v31i4.18

106.	Kassaw MW, Abebe AM, Tegegne KD, Getu MA, Bihonegn WT. Prevalence and associations of active trachoma among rural preschool children in Wadla district, northern Ethiopia. BMC Ophthalmol. 26 de dezembro de 2020;20(1):346. doi:10.1186/s12886-020-01585-9

107.	Ngondi J, Ole-Sempele F, Onsarigo A, Matende I, Baba S, Reacher M, et al. Blinding Trachoma in Postconflict Southern Sudan. PLoS Med. 19 de dezembro de 2006;3(12):e478. doi:10.1371/journal.pmed.0030478

108.	Frick KD. Trichiasis and Disability in a Trachoma-Endemic Area of Tanzania. Archives of Ophthalmology. 1o de dezembro de 2001;119(12):1839. doi:10.1001/archopht.119.12.1839

109.	Kortlang C, Koster JCA, Coulibaly S, Dubbeldam RP. Prevalence of blindness and visual impairment in the region of Ségou, Mali. A baseline survey for a primary eye care programme. Tropical Medicine & International Health. 3 de junho de 1996;1(3):314–9. doi:10.1046/j.1365-3156.1996.d01-44.x

110.	Irawati Y, Andayani G, Rahayu T, Zakiyah H, Kurniawardhani DR, Paramita C, et al. Vision-related quality of life among released from treatment cases of leprosy evaluated with NEI-VFQ-25: a cross-sectional study. BMC Ophthalmol. 2 de outubro de 2023;23(1):398. doi:10.1186/s12886-023-03146-2

111.	Bispo AJB, Almeida MLD, de Almeida RP, Bispo Neto J, de Oliveira Brito AV, França CM. Pulmonary involvement in human visceral leishmaniasis: Clinical and tomographic evaluation. PLoS One. 30 de janeiro de 2020;15(1):e0228176. doi:10.1371/journal.pone.0228176

112.	Singh L, Malhotra R, Bundela R, Garg P, Dhillon K, Chawla S, et al. Ocular Disability - WHO Grade 2 in persons affected with leprosy. Indian J Lepr. 2014;86:1–6.

113.	Kayembe DL, Kasonga DL, Kayembe PK, Mwanza J ‐C. K, Boussinesq M. Profile of eye lesions and vision loss: a cross‐sectional study in Lusambo, a forest–savanna area hyperendemic for onchocerciasis in the Democratic Republic of Congo. Tropical Medicine & International Health. 16 de janeiro de 2003;8(1):83–9. doi:10.1046/j.1365-3156.2003.00957.x

114.	Dana MR. Ocular Manifestations of Leprosy in a Noninstitutionalized Community in the United States. Archives of Ophthalmology. 1o de maio de 1994;112(5):626. doi:10.1001/archopht.1994.01090170070025

115.	Rawlani SS, Rawlani SM. Evaluation of hearing impairment in leprosy patients taking multidrug therapy. J Anat Soc India. setembro de 2016;65:S51–2. doi:10.1016/j.jasi.2016.08.168

116.	Reis MC, Raposo MT, Alencar CHM de, Ramos Júnior AN, Heukelbach J. Incapacidades físicas em pessoas que concluíram a poliquimioterapia para hanseníase em Vitória da Conquista, Bahia, Brasil. Acta Fisiátrica. 30 de junho de 2018;25(2):78–85. doi:10.11606/issn.2317-0190.v25i2a162582

117.	Jayawardana S, Arambepola C, Chang T, Gnanathasan A. Long-term health complications following snake envenoming. J Multidiscip Healthc. junho de 2018;Volume 11:279–85. doi:10.2147/JMDH.S126648

118.	Williams SS, Wijesinghe CA, Jayamanne SF, Buckley NA, Dawson AH, Lalloo DG, et al. Delayed Psychological Morbidity Associated with Snakebite Envenoming. PLoS Negl Trop Dis. 2 de agosto de 2011;5(8):e1255. doi:10.1371/journal.pntd.0001255

119.	Véras GCB, Silva LH da, Sarmento WM, Moraes RM de, Oliveira SH dos S, Soares MJGO. Características sociodemográficas e epidemiológicas relacionadas ao grau de incapacidade física em hanseníase no estado da Paraíba, Brasil. Hansenologia Internationalis: hanseníase e outras doenças infecciosas. 18 de julho de 2023;48:1–15. doi:10.47878/hi.2023.v48.38999

120.	Pescarini JM, Teixeira CSS, Silva NB da, Sanchez MN, Natividade MS da, Rodrigues LC, et al. Epidemiological characteristics and temporal trends of new leprosy cases in Brazil: 2006 to 2017. Cad Saude Publica. 2021;37(7). doi:10.1590/0102-311x00130020

121.	Albuquerque AR de, Silva JV de M, Barreto E de O, Fraga CA de C, Santos WO dos, Silva MSM da, et al. Epidemiological, temporal and spatial dynamics of leprosy in a municipality in northeastern Brazil (2008-2017): an ecological study. Rev Soc Bras Med Trop. 2020;53. doi:10.1590/0037-8682-0246-2020

122.	Souza CDF de, Tavares DL de C, Tavares CM, Almeida AGC dos S, Accioly SMP da S, Paiva JPS de, et al. Physical disabilities due to leprosy in Alagoas State, Northeast Brazil: a temporal and spatial modeling. Rev Soc Bras Med Trop. 2019;52. doi:10.1590/0037-8682-0540-2018

123.	Pereira KC, Bueno IDC, Lana FCF. TENDÊNCIA EPIDEMIOLÓGICA DA HANSENÍASE EM MINAS GERAIS (1995-2015). Cogitare Enfermagem. 12 de dezembro de 2019;24. doi:10.5380/ce.v24i0.66109

124.	Souza CDF de, Fernandes TRM de O, Matos TS, Ribeiro Filho JM, Almeida GKA de, Lima JCB, et al. Physical disability degree in the elderly population affected by leprosy in the state of Bahia, Brazil. Acta Fisiátrica. 2017;24(1). doi:10.5935/0104-7795.20170006

125.	Vieira CS de CA, Mariano MLL, Silva EM. A PREVALÊNCIA DAS DEFICIÊNCIAS FÍSICAS DA HANSENÍASE APÓS ALTA MEDICAMENTOSA EM MUNICÍPIOS QUE ALCANÇARAM A META DE ELIMINAÇÃO . Hansenol Int. 2016;41:46–54.

126.	Oliveira TAP de, Carvalho CL, Galiciolli R, Santangelo EM, Souza RA de. ESTUDO DAS INCAPACIDADES DOS CASOS NOTIFICADOS DE HANSENÍASEEM UMA GERÊNCIA REGIONAL DE SAÚDE DO VALE DO JEQUITINHONHA ENTRE 2001 E 2008. Hansenol Int. 2010;35(1):45–52.

127.	Lana FCF, Amaral EP, Lanza FM, Saldanha AN e SL de. Physical disabilities resulting from hansen’s disease in Vale do Jequitinhonha/State of Minas Gerais, Brazil. Rev Lat Am Enfermagem. dezembro de 2008;16(6):993–7. doi:10.1590/S0104-11692008000600009

128.	Silva Sobrinho RA da, Mathias TA de F, Gomes EA, Lincoln PB. Evaluation of incapacity level in leprosy: a strategy to sensitize and train the nursing team. Rev Lat Am Enfermagem. dezembro de 2007;15(6):1125–30. doi:10.1590/S0104-11692007000600011

129.	Duarte MTC, Ayres JA, Simonetti JP. Socioeconomic and demographic profile of leprosy carriers attended in nursing consultations. Rev Lat Am Enfermagem. outubro de 2007;15(spe):774–9. doi:10.1590/S0104-11692007000700010

130.	Amorim D de S, Marin-Neto JA. Alteraçöes funcionais do sistema nervoso autônomo na doença de Chagas. Revista da Sociedade de Cardiologia do Estado de São Paulo. 1994;4.

131.	Al Faran MF. Low prevalence of trachoma in the South Western part of Saudi Arabia, results of a population based study. Int Ophthalmol. 1995;18(6):379–82. doi:10.1007/BF00930319

132.	Petrela R, Kuneshka L, Foto E, Zavalani F, Gradoni L. Pediatric Visceral Leishmaniasis in Albania: A Retrospective Analysis of 1,210 Consecutive Hospitalized Patients (1995–2009). PLoS Negl Trop Dis. 7 de setembro de 2010;4(9):e814. doi:10.1371/journal.pntd.0000814

133.	Jayawardana S, Gnanathasan A, Arambepola C, Chang T. Chronic Musculoskeletal Disabilities following Snake Envenoming in Sri Lanka: A Population-Based Study. PLoS Negl Trop Dis. 4 de novembro de 2016;10(11):e0005103. doi:10.1371/journal.pntd.0005103

134.	Aglanu LM, Amuasi JH, Schut BA, Steinhorst J, Beyuo A, Dari CD, et al. What the snake leaves in its wake: Functional limitations and disabilities among snakebite victims in Ghanaian communities. PLoS Negl Trop Dis. 23 de maio de 2022;16(5):e0010322. doi:10.1371/journal.pntd.0010322

135.	Suman AC, Costa ÉAPN da, Bazan SGZ, Hueb JC, Carvalho FC de, Martin LC, et al. Evaluating respiratory musculature, quality of life, anxiety, and depression among patients with indeterminate chronic Chagas disease and symptoms of pulmonary hypertension. Rev Soc Bras Med Trop. março de 2017;50(2):194–8. doi:10.1590/0037-8682-0198-2016

136.	Bentes GL, Matayoshi S, Talhari C. Lagophthalmos in leprosy: clinical experience in an Amazon reference center. Rev Bras Oftalmol. 2021;80(1). doi:10.5935/0034-7280.20210005

137.	Souza CDF de, Luna CF, Magalhães M de AFM. Spatial modeling of leprosy in the state of Bahia and its social determinants: a study of health inequities. An Bras Dermatol. 9 de maio de 2019;94(2):182–91. doi:10.1590/abd1806-4841.20197554

138.	Santana EMF de, Brito KKG de, Nogueira JDA, Leadebal ODCP, Costa MML, Silva MA da, et al. Deficiências e incapacidades na hanseníase: do diagnóstico à alta por cura. Revista Eletrônica de Enfermagem. 27 de agosto de 2018;20. doi:10.5216/ree.v20.50436

139.	Benedicto CB, Marques T, Milano AP, Galan NG de A, Nardi ST, Duerksen F, et al. Avaliação da qualidade de vida, grau de incapacidade e do desenho da figura humana em pacientes com neuropatias na hanseníase. Acta Fisiátrica. 2018;24(3). doi:10.5935/0104-7795.20170022

140.	Queirós MI, Ramos Júnior AN, Alencar CHM, Monteiro LD, Sena AL, Barbosa JC. Clinical and epidemiological profile of leprosy patients attended at Ceará, 2007-2011. An Bras Dermatol. junho de 2016;91(3):311–7. doi:10.1590/abd1806-4841.20164102

141.	Galan NG de A, Beluci ML, Marciano LHSC, Ruiz RBP, Oliveira NGG de, Bonini AG, et al. AVALIAÇÃO DA PRÁTICA DO AUTOCUIDADO DOMICILIAR EM HANSENÍASE. Hansenol Int. 2016;41(1):37–45.

142.	Oliveira DT de, Sherlock J, Melo EV de, Rollemberg KCV, Paixao TRS da, Abuawad YG, et al. Clinical variables associated with leprosy reactions and persistence of physical impairment. Rev Soc Bras Med Trop. 21 de outubro de 2013;46(5):600–4. doi:10.1590/0037-8682-0100-2013

143.	Kil AKA, Silvestre CM, Kaminice LM, Quintino LB, Lima LB de, Paranhos MB, et al. DEFICIÊNCIAS E INCAPACIDADES POR HANSENÍASE: AVALIAÇÃO CLÍNICA E EPIDEMIOLÓGICA DOS PACIENTES ATENDIDOS EM UM CENTRO DE REFERÊNCIA NACIONAL DO BRASIL. Hansenologia Internationalis . 2012;37(1):25–33.

144.	Julio MVRF, Nardi SMT, Pedro H da SP, Paschoal VD. EVOLUÇÃO DAS LESÕES NASAIS EM PACIENTES COM HANSENÍASE. Hansenologia Internationalis . 2010;35(1):29–35.

145.	Araújo KCG, Silva C da R e, Barbosa CS, Ferrari TC. Clinical-epidemiological profile of children with schistosomal myeloradiculopathy attended at the Instituto Materno-Infantil de Pernambuco. Mem Inst Oswaldo Cruz. outubro de 2006;101(suppl 1):149–56. doi:10.1590/S0074-02762006000900024

146.	Mercado M, Acosta-Reyes J, Parra E, Guzmán L, Beltrán M, Gasque P, et al. Renal involvement in fatal cases of chikungunya virus infection. Journal of Clinical Virology. junho de 2018;103:16–8. doi:10.1016/j.jcv.2018.03.009

147.	Abiose A, Murdoch I, Babalola O, Cousens S, Liman I, Onyema J, et al. Distribution and aetiology of blindness and visual impairment in mesoendemic onchocercal communities, Kaduna State, Nigeria. Kaduna Collaboration for Research on Onchocerciasis. British Journal of Ophthalmology. 1o de janeiro de 1994;78(1):8–13. doi:10.1136/bjo.78.1.8

148.	Akogun OB. Eye lesions, blindness and visual impairment in the Taraba river valley, Nigeria and their relation to onchocercal microfilariae in skin. Acta Trop. junho de 1992;51(2):143–9. doi:10.1016/0001-706X(92)90056-4

149.	Newland HS, White AT, Greene BM, Murphy RP, Taylor HR. Ocular manifestations of onchocerciasis in a rain forest area of west Africa. British Journal of Ophthalmology. 1o de março de 1991;75(3):163–9. doi:10.1136/bjo.75.3.163

150.	Brenes-Chacon H, Gutierrez JM, Camacho-Badilla K, Soriano-Fallas A, Ulloa-Gutierrez R, Valverde K, et al. Long-term sequelae secondary to snakebite envenoming: a single centre retrospective study in a Costa Rican paediatric hospital. BMJ Paediatr Open. 16 de setembro de 2020;4(1):e000735. doi:10.1136/bmjpo-2020-000735

151.	Silva FS, Ibiapina HNS, Neves JCF, Coelho KF, Barbosa FBA, Lacerda MVG, et al. Severe tissue complications in patients of Bothrops snakebite at a tertiary health unit in the Brazilian Amazon: clinical characteristics and associated factors. Rev Soc Bras Med Trop. 2021;54. doi:10.1590/0037-8682-0374-2020

152.	Kuper H. Neglected tropical diseases and disability—what is the link? Trans R Soc Trop Med Hyg. 1o de dezembro de 2019;113(12):839–44. doi:10.1093/trstmh/trz001

153.	Agrawal A, Pandit L, Dalal M, Shetty JP. Neurological manifestations of Hansen’s disease and their management. Clin Neurol Neurosurg. outubro de 2005;107(6):445–54. doi:10.1016/j.clineuro.2005.03.007

154.	Chen X, Di L, Qian M, Shen D, Feng X, Zhang X. Neurological features of Hansen disease: a retrospective, multicenter cohort study. Sci Rep. 6 de maio de 2024;14(1):10374. doi:10.1038/s41598-024-60457-0

155.	Kuper H. Disability, mental health, stigma and discrimination and neglected tropical diseases. Trans R Soc Trop Med Hyg. 28 de janeiro de 2021;115(2):145–6. doi:10.1093/trstmh/traa160

156.	Sanchez MN, Nery JS, Pescarini JM, Mendes AA, Ichihara MY, Teixeira CSS, et al. Physical disabilities caused by leprosy in 100 million cohort in Brazil. BMC Infect Dis. 22 de dezembro de 2021;21(1):290. doi:10.1186/s12879-021-05846-w

157.	Wang EY, Kong X, Wolle M, Gasquet N, Ssekasanvu J, Mariotti SP, et al. Global Trends in Blindness and Vision Impairment Resulting from Corneal Opacity 1984–2020. Ophthalmology. agosto de 2023;130(8):863–71. doi:10.1016/j.ophtha.2023.03.012

158.	Nunes MCP, Beaton A, Acquatella H, Bern C, Bolger AF, Echeverría LE, et al. Chagas Cardiomyopathy: An Update of Current Clinical Knowledge and Management: A Scientific Statement From the American Heart Association. Circulation. 18 de setembro de 2018;138(12). doi:10.1161/CIR.0000000000000599

159.	van Wijk R, van Selm L, Barbosa MC, van Brakel WH, Waltz M, Philipp Puchner K. Psychosocial burden of neglected tropical diseases in eastern Colombia: an explorative qualitative study in persons affected by leprosy, cutaneous leishmaniasis and Chagas disease. Global Mental Health. 18 de junho de 2021;8:e21. doi:10.1017/gmh.2021.18

160.	Fischer J, Jansen B, Rivera A, Gómez LJ, Barbosa MC, Bilbao JL, et al. Validation of a cross-NTD toolkit for assessment of NTD-related morbidity and disability. A cross-cultural qualitative validation of study instruments in Colombia. PLoS One. 3 de dezembro de 2019;14(12):e0223042. doi:10.1371/journal.pone.0223042

161.	Almeida-Val F, Goulart C da L, Monteiro WM, Tinoco Areas GP. When Surviving Neglected Tropical Diseases Is Not Enough. J Infect Dis. 17 de março de 2025;231(3):822–3. doi:10.1093/infdis/jiae579

**Figures**

**Figure 1. PRISMA flowchart of study selection.**

Flow diagram showing the number of records identified, screened, excluded, and finally included in the systematic review.

**Figure 2. Systems and organs affected by disabilities related to neglected tropical diseases.**

Distribution of sequelae reported in the included studies, grouped by affected system (neurological, musculoskeletal, visual, cardiovascular, gastrointestinal, psychosocial, and others).

**Figure 3. Map showing the location of study samples.**

Distribution of studies reporting sequelae included in systematic review.

**Tables**

| **Table 1. Characteristics of the studies included in the systematic review** | | | | | | |
| --- | --- | --- | --- | --- | --- | --- |
| **#** | **First author, Year** |  | **Female sex, n (%)** | **Age, mean ± SD** | **Geographical origin** | **Neglected**  **disease** |
| *Case report* |  |  |  |  |  |  |
| 1 | Pires, 2020 (22) | 1 | 0 (0) | 36 | Brazil | Leprosy |
| 2 | Parente, 2018 (23) | 1 | 1 (100) | 55 | Brazil | Leprosy |
| 3 | Ortega, 2018 (24) | 1 | 0 (0) | 51 | Colombia | Neurocysticercosis |
| 4 | Diaz-Ramirez, 2017 (25) | 1 | 0 (0) | 25 | Colombia | Leprosy |
| 5 | Dametto, 2016 (26) | 1 | 0 (0) | 24 | Brazil | Neurocysticercosis |
| 6 | Hernández-Beltrán, 2015 (27) | 1 | 1 (100) | 26 | Colombia | Chagas disease |
| 7 | Seijo, 2014 (28) | 1 | 0 (0) | 38 | Argentina | Chikungunya |
| 8 | Rigo, 2008 (29) | 1 | 0 (0) | 64 | Brazil | Leishmaniasis |
| 9 | Filho, 2007 (30) | 3 | 1 (33) | 25 ± 10 | Brazil | Schistosomiasis |
| 10 | Parajuli, 2020 (31) | 1 | 0 (0) | 67 | Nepal | Leishmaniasis |
| 11 | Pradhan, 2020 (32) | 1 | 1 (100) | 37 | Bhutan | Leishmaniasis |
| 12 | Pallangyo, 2020 (33) | 1 | 0 (0) | 38 | Tanzania | Schistosomiasis |
| 13 | Dalugama, 2018 (34) | 1 | 1 (100) | 43 | Sri Lanka | Dengue |
| 14 | Sadiq, 2014 (35) | 1 | 1 (100) | 50 | Paquistan | Dengue |
| 15 | Chang, 2007 (36) | 1 | 1 (100) | 32 | China | Dengue |
| 16 | Yan, 2006 (37) | 1 | 0 (0) | 31 | Singapore | Dengue |
| 17 | Bentes, 2024 (38) | 1 | 1 (100) | 54 | Brazil | Snakebite |
| 18 | Susmitha, 2024 (39) | 1 | 0 (0) | 33 | India | Snakebite |
| *Case series* |  |  |  |  |  |  |
| 19 | Rhee, 2014 (40) | 3 | 3 (100) | 36 ± 6 | South Korea | Dengue |
| 20 | Koh, 2013 (41) | 11 | 4 (36,4) | 27.3 | Singapore | Dengue |
| 21 | Espiritu, 1991 (42) | 4 | 1 (25) | NR | USA | Leprosy |
| *Case-Control* |  |  |  |  |  |  |
| 22 | Pazin-Filho, 2006 (43) | 59 | NR | 55.0 | Brazil | Chagas disease |
| 23 | Dantas, 2002 (44) | 81 | 50 (61,7) | NR | Brazil | Chagas disease |
| 24 | Mangone, 1994 (45) | 45 | 26 (57,7) | 36.9 ± 10.5 | Argentina | Chagas disease |
| 25 | Zicker, 1988 (46) | 120 | 56 (47) | 43.9 | Brazil | Chagas disease |
| *Clinical Trial* | |  |  |  |  |  |
| 26 | Barda, 2017 (47) | 303 | 168 (55,4) | 6.2 | Switzerland | Schistosomiasis |
| 27 | Masud, 2008 (48) | 240 | 83 (34,5) | 34 | Liberia | Onchocerciasis |
| 28 | Wijesinghe, 2015 (49) | 225 | 157 (69,7) | 42.1 ± 12.4 | Sri Lanka | Snakebite |
| 29 | Melese, 2005 (50) | 1452 | 1121 (77) | 50 | United Kingdom | Trachoma |
| 30 | Agbenorku, 2011 (51) | 38 | 15 (39.4) | 14 | Ghana | Burili Ulcer |
| *Cohort* |  |  |  |  |  |  |
| 31 | Duvignaud, 2018 (52) | 22 | 20 (89.6) | 46.4 ± 12.0 | England | Chikungunya |
| 32 | Ribeiro, 2015 (53) | 107 | 55 (52,1) | NR | Brazil | Leprosy |
| 33 | Borges-Pereira, 1998 (54) | 298 | 183 (61,4) | 50.0 | Brazil | Chagas disease |
| 34 | Bowman, 2001 (55) | 326 | 208 (64) | 43 ± 21 | United Kingdom | Trachoma |
| *Cross-sectional* | |  |  |  |  |  |
| 35 | Bernardes, 2009 (56) | 69 | 28 (40) | NR | Brazil | Leprosy |
| 36 | Klis, 2014 (57) | 127 | 86 (67,7) | 18.0 | Netherlands | Burili Ulcer |
| 37 | Costa, 2023 (58) | 24 | 3 (12,5) |  | Brazil | Leprosy |
| 38 | Bomtempo, 2023 (59) | 71 | 38 (53,5) | 46.0 | Brazil | Leprosy |
| 39 | Machado, 2022 (60) | 37 | 37 (100) | 53.45 ± 7.32 | Brazil | Chicungunya |
| 40 | Chaves, 2022 (61) | 392 | 141 (35,9) | NR | Brazil | Leprosy |
| 41 | Matos, 2021 (62) | 50 | 25 (50) | 0 a >60 anos | Brazil | Leprosy |
| 42 | D'Azevedo, 2021 (63) | 43 | 20 (46,5) | NR | Brazil | Leprosy |
| 43 | Campos, 2020 (64) | 101 | 66 (65,3) | NR | Brazil | Chagas disease |
| 44 | Sobral, 2020 (65) | 42 | 6 (14,2) | NR | Brazil | Leprosy |
| 45 | Abella, 2019 (66) | 94 | 76 (80,8) | 57 | Colombia | Chikungunya |
| 46 | Silva, 2019 (67) | 323 | NR | NR | Brazil | Leprosy |
| 47 | Robinet, 2019 (68) | 14 | 2 (14,2) | NR | Cuba | Leprosy |
| 48 | Silva, 2018 (69) | 323 | 134 (41,4) | 37.7 | Brazil | Leprosy |
| 49 | Loiola, 2018 (70) | 40 | 18 (45) | NR | Brazil | Leprosy |
| 50 | da Silva, 2018 (71) | 896 | 444 (49,5) | NR | Brazil | Leprosy |
| 51 | Aben-Athar, 2017 (72) | 84 | 30 (35,7) | NR | Brazil | Leprosy |
| 52 | Basso, 2017 (73) | 52 | 12 (23) | NR | Brazil | Leprosy |
| 53 | Belkiman-Pedro, 2018 (74) | 50 | 9 (18) | 57,06 | Brazil | Leprosy |
| 54 | Del Arco, 2016 (75) | 22 | 14 (63,6) | 51 ± 10.78 | Brazil | Leprosy |
| 55 | Gaudenci, 2016 (76) | 32 | 13 (40,6) | 49.3 | Brazil | Leprosy |
| 56 | Monteiro, 2014 (77) | 282 | 137 (48,5) | 45.8 | Brazil | Leprosy |
| 57 | Pelarigo, 2014 (78) | 90 | 22 (24,4) | 68,91 ± 7.3 | Brazil | Leprosy |
| 58 | Carvalho, 2014 (79) | 118 | 30 (25,4) | 41.9 | Brazil | Leprosy |
| 59 | Araújo, 2014 (80) | 1770 | 851 (48) | 57 ± 4.5 | Brazil | Leprosy |
| 60 | Monteiro, 2013 (81) | 282 | 137 (48,6) | 45.8 | Brazil | Leprosy |
| 61 | Carvalho, 2015 (82) | 26 | 11 (42,3) | 62 | Brazil | Leprosy |
| 62 | Baldan, 2012 (83) | 50 | 29 (58) | 47 ± 7.8 | Brazil | Leprosy |
| 63 | Pieri, 2014 (84) | 245 | 116 (47,3) | 46 | Brazil | Leprosy |
| 64 | Silva, 2012 (85) | 69 | 43 (62,3) | NR | Brazil | Leprosy |
| 65 | Guerrero, 2013 (86) | 333 | 121 (36,3) | 49.1 | Colombia | Leprosy |
| 66 | Budel, 2011 (87) | 22 | 10 (45) | 50.2 | Brazil | Leprosy |
| 67 | Seijo, 2011 (88) | 13 | 0 (0) | 38 ± 14 | Argentina | St. Louis Encephalitis |
| 68 | Raposo, 2011 (89) | 61 | 32 (52,5) | 41 | Brazil | Leprosy |
| 69 | de Sousa, 2011 (90) | 100 | 46 (46) | 39.2 | Brazil | Leprosy |
| 70 | Nardi, 2011 (91) | 384 | NR | 51,7 ± 15,18 | Brazil | Leprosy |
| 71 | Rodini, 2010 (92) | 26 | 11 (42,3) | 51 | Brazil | Leprosy |
| 72 | Silva, 2008 (93) | 417 | 126 (30,2) | NR | Brazil | Chagas disease |
| 73 | Barbosa, 2008 (94) | 69 | 43 (62,3) | 46 | Brazil | Leprosy |
| 74 | Cunha, 2009 (95) | 158 | 22 (13,9) | >15 - <55 | Brazil | Leprosy |
| 75 | Goldschmidt, 2007 (96) | 941 | NR | NR | Mexico | Chlamydia trachomatis |
| 76 | Escarabel, 2008 (97) | 60 | 12 (20) | 7 ± 3.4 | Brazil | Leprosy |
| 77 | Carvalho, 2000 (98) | 81 | 43 (53) | NR | Brazil | Leprosy |
| 78 | Maradel, 1998 (99) | 300 | 95 (31,7) | 58.6 | Brazil | Leprosy |
| 79 | Mol, 2023 (100) | 62 | 39 (62,9) | NR | Netherlands | Leprosy |
| 80 | Vêncio, 1988 (101) | 348 | NR | NR | Brazil | Snakebite |
| 81 | Baranwal, 2020 (102) | 2150 | 891 (41,4) | NR | India | Onchocerciasis |
| 82 | Pedrazzanie, 1985 (103) | 160 | NA | NR | Brazil | Leprosy |
| 83 | Olamiju, 2023 (104) | 158 | 53 (33,5) | NR | Nigeria | Onchocerciasis |
| 84 | Getahun, 2021 (105) | 778 | 400 (51,4) | 11.3 | Ethiopia | Onchocerciasis |
| 85 | Kassaw, 2020 (106) | 596 | 292 (49) | NR | Ethiopia | Trachoma |
| 86 | Ngondi, 2006 (107) | 3567 | 1979 (55,5) | 17.6 | Sudan | Trachoma |
| 87 | Frick, 2001 (108) | 3064 | 1593 (55,2) | NR | Tanzania | Trachoma |
| 88 | Kortlang, 1996 (109) | 5871 | 3063 (52,1) | NR | Mali | Trachoma |
| 89 | Yunia, 2023 (110) | 325 | 119 (36,6) | 53.2 | United Kingdom | Leprosy |
| 90 | Bispo, 2020 (111) | 42 | 14 (33,3) | 19.5 | USA | Leishmaniasis |
| 91 | Singh, 2014 (112) | 302 | 76 (25,1) | 36 | India | Leprosy |
| 92 | Kayembe, 2003 (113) | 750 | 357 (47,6) | 40 | Republic of Congo | Onchocerciasis |
| 93 | Dana, 1994 (114) | 61 | 18 (29,5) | NR | USA | Leprosy |
| 94 | Rawlany, 2013 (115) | 30 | 15 (50) | 46.3 | India | Leprosy |
| 95 | Reis, 2018 (116) | 222 | 108 | NR | Brazil | Leprosy |
| 96 | Jayawardana, 2018 (117) | 112 | 49 (44) | 42.8 ± 12.4 | Sri Lanka | Snakebite |
| 97 | Williams, 2011 (118) | 88 | 14 (16) | 41.6 ± 11.8 | Sri Lanka | Snakebite |
| *Ecological* |  |  |  |  |  |  |
| 98 | Véras, 2023 (119) | 1900 | 869 (45,7) | NR | Brazil | Leprosy |
| 99 | Pescarini, 2021 (120) | 396989 | 177646 (44,7) | <15 anos / >15 | Brazil | Leprosy |
| 100 | Albuquerque, 2020 (121) | 292 | 151 (51,7) | NR | Brazil | Leprosy |
| 101 | Souza, 2019 (122) | 4252 | NR | NR | Brazil | Leprosy |
| 102 | Pereira, 2019 (123) | 50673 | NR | NR | Brazil | Leprosy |
| 103 | de Souza, 2017 (124) | 5973 | 2882 (48,2) | NR | Brazil | Leprosy |
| 104 | Vieira, 2016 (125) | 126 | 70 (55,5) | 56.02 ± 15.71 | Brazil | Leprosy |
| 105 | Oliveira, 2010 (126) | 542 | 278 (51,2) | 42.5 ± 17.2 | Brazil | Leprosy |
| 106 | Lana, 2008 (127) | 1456 | 711 (48,8) | NR | Brazil | Leprosy |
| 107 | Sobrinho, 2007 (128) | 99 | 32 (32,3) | 50 ± 8.8 | Brazil | Leprosy |
| 108 | Duarte, 2007 (129) | 37 | 16 (43,2) | 42 ± 3.3 | Brazil | Leprosy |
| 109 | de Souza, 2006 (130) | 187 | 78 (42.2) | NR | Brazil | Chagas disease |
| 110 | Al Faran, 2994 (131) | 19 | NR | NR | Saudi Arabia | Trachoma |
| 111 | Petrela, 2010 (132) | 1210 | 508 (42) | 8 ± 3.1 | USA | Leishmaniasis |
| 112 | Jayawardana, 2016 (133) | 816 | 326 (40) | 42.7 ± 16.3 | Sri Lanka | Snakebite |
| 113 | Aglanu, 2022 (134) | 193 | 98 (50,8) | 37 ± 9.7 | Gana | Snakebite |
| *Prospective* |  |  |  |  |  |  |
| 114 | Suman, 2017 (135) | 107 | 49 (45,7) | 57 | Brazil | Chagas disease |
| *Retrospective* |  |  |  |  |  |  |
| 115 | Bentes, 2021 (136) | 65 | 43 (66,1) | NR | Brazil | Leprosy |
| 116 | Souza, 2019 (137) | 42227 | NR | NR | Brazil | Leprosy |
| 117 | Santana, 2018 (138) | 414 | 211 (51) | NR | Brazil | Leprosy |
| 118 | Benedicto, 2018 (139) | 100 | 66 (66) | NR | Brazil | Leprosy |
| 119 | Queirós, 2016 (140) | 475 | 242 (51,8) | 45,2 | Brazil | Leprosy |
| 120 | Galan, 2016 (141) | 11 | 2 (18,1) | NR | Brazil | Leprosy |
| 121 | de Oliveira, 2013 (142) | 494 | 227 (46) | NR | Brazil | Leprosy |
| 122 | Kil, 2012 (143) | 318 | NR | 38 | Brazil | Leprosy |
| 123 | Julio, 2010 (144) | 212 | NR | 46.6 ± 16.2 | Brazil | Leprosy |
| 124 | Araújo, 2006 (145) | 10 | 3 (30) | 7.3 | Brazil | Schistosomiasis |
| 125 | Mercado, 2018 (146) | 13 | 3 (23) | NR | Netherlands | Chikungunya |
| 126 | Abiose, 1994 (147) | 6831 | NR | NR | Nigeria | Onchocerciasis |
| 127 | Akogun, 1992 (148) | 2876 | 1314 (45,6) | NR | Nigeria | Onchocerciasis |
| 128 | Newland, 1991 (149) | 800 | 288 (36,1) | NR | Africa | Onchocerciasis |
| 129 | Brenes-Chacon, 2020 (150) | 74 | 22 (29,7) | NR | Costa Rica | Snakebite |
| 130 | Silva, 2021 (151) | 307 | 65 (21) | 38.6 ± 14.5 | Brazil | Snakebite |
| NR: Not reported | | | | | | |

| **Table 2. Comorbidities and sequelae reported by the populations evaluated in the included studies** | |
| --- | --- |
| Total evaluated | N = 551574 |
| *Reported comorbidities* |  |
| Respiratory diseases (%, n) | 0,2 (n=1019) |
| Heart diseases (%, n) | 0,1 (n=540) |
| Anxiety/Depression (%, n) | 0,05 (n=295) |
| Hypertension (%, n) | 0,03 (n=180) |
| Diabetes (%, n) | 0,02 (n=149) |
| Obesity (%, n) | 0,01 (n=87) |
| *Reported Sequelae* |  |
| Physical/motor disabilities (%, n) | 24,6 (n=135683) |
| Visual deficits (%, n) | 1,81 (n=10007) |
| Skin problems (%, n) | 0,33 (n=1816) |
| Gastrointestinal deficits (%, n) | 0,25 (n=1379) |
| Fever (%, n) | 0,24 (n=1319) |
| Appetite changes (%, n) | 0,19 (n=1034) |
| Abdominal pain (%, n) | 0,18 (n=985) |
| Nerve injuries (%, n) | 0,17 (n=964) |
| Motor dysfunctions (%, n) | 0,15 (n=803) |
| Fatigue (%, n) | 0,12 (n=667) |
| Paresthesia (%, n) | 0,10 (n=538) |
| Dyspnea (%, n) | 0,09 (n=437) |
| Cough (%, n) | 0,07 (n=373) |
| Muscle pain (%, n) | 0,06 (n=304) |
| Arrhythmias/tachycardias (%, n) | 0,05 (n=273) |
| Joint pain (%, n) | 0,04 (n=218) |
| Urinary changes (%, n) | 0,03 (n=159) |
| Depression (%, n) | 0,03 (n=158) |
| Psychological disorders (%, n) | 0,02 (n=135) |
| Emotional impacts in work (%, n) | 0,02 (n=127) |
| Headache (%, n) | 0,02 (n=112) |
| Limb edema (%, n) | 0,02 (n=110) |
| Hypertension (%, n) | 0,02 (n=85) |
| Chest pain (%, n) | 0,01 (n=66) |
| Dysphagia (%, n) | 0,01 (n=57) |
| Hearing problems (%, n) | 0,01 (n=45) |
| Emotional difficulties in family life (%, n) | 0,01 (n=42) |
| Muscle weakness (%, n) | 0,01 (n=40) |
| Deficiency in social life (%, n) | 0,01 (n=37) |
| Anxiety (%, n) | 0,01 (n=36) |
| Mental confusion (%, n) | 0,004 (n=26) |
| Sleep problems (%, n) | 0,004 (n=23) |
| Memory problems (%, n) | 0,003 (n=21) |
| Cognitive deficits (%, n) | 0,003 (n=18) |
| Voice/speech changes (%, n) | 0,001 (n=7) |

**Figure 1. PRISMA flowchart of study selection.**

**Identification of studies via databases and registers**

Records identified from:

Medline/ PubMed; Scielo; Lilacs, Chochrane, CINAHL, Embase and Web of Science.

Databases (n =7)

Registers (n = 958)

Records removed *before screening*:

Duplicate records removed

(n = 478)

**Identification**

Records excluded (n = 179)

Not human studies (n = 132)

Grey literature (n = 47)

Records screened

(n = 480)

**Screening**

Reports excluded: (n = 180)

Reviews, Editorials, Letters, Short Reports and Comments (n = 105)

Laboratory or in vitro studies (n = 44)

Without NTD (n = 11)

Without mentions of sequelae (n = 20).

Reports assessed for eligibility

(n = 310)

Studies included in review

(n = 130)

**Included**

**[Figure 2. Systems and organs affected by disabilities related to neglected tropical diseases].**

**[Figure 3. Map showing the location of the study samples].**
