# Supplementary material for: Disability as a neglected outcome of neglected tropical diseases: A systematic review
Source: PLoS Negl Trop Dis. 2026 Jul 1;20(7):e0014480. doi: 10.1371/journal.pntd.0014480 (PMC13345467; doi:10.1371/journal.pntd.0014480)
Supplement: Attachment — Submitted filename: (update) response to the reviewers.docx [file pntd.0014480.s003.docx]

Response to Reviewers (UPDATE)

Manuscript ID: PNTD-D-26-00064R1

Title: Disability as a neglected outcome of neglected tropical diseases: a systematic review

We would like to thank the Editor and Reviewers for their careful evaluation of our manuscript and for their constructive comments. We have revised the manuscript accordingly and believe that the changes have substantially improved its clarity, transparency, and reproducibility. All modifications have been highlighted in the revised manuscript.

**Response to the Academic Editor**

Comment:
Please clarify the languages in which search terms were searched for, considering the high number of Brazilian and Spanish-language studies included.

Response:
Thank you for this observation. We have revised the search strategy and explicitly clarified in the Methods section that equivalent search terms in Portuguese and Spanish were included in addition to English descriptors. The complete updated search strategy has been provided in the revised manuscript and supplementary materials. This modification better reflects the multilingual approach used to identify studies published in Latin America and other endemic regions.

**Response to Reviewer #1**

Comment:
The research question itself is still missing.

Response:
We appreciate this comment. To improve methodological clarity, we revised the Methods section and explicitly stated the research question guiding the review. The research question is now clearly presented.

Comment:
Given the higher number of Brazilian studies and the declaration that no language restrictions were applied, it is strange that only English MeSH terms were presented.

Response:
We agree that the previous version did not adequately describe the multilingual search strategy. The search strategy has been revised to explicitly include Portuguese and Spanish terms corresponding to both neglected tropical diseases and disability-related outcomes. This clarification has been added to the Methods section and supplementary search strategy.

Comment:
The number of studies in each step of the PRISMA flow chart should be revised.

Response:
Thank you for identifying this inconsistency. The PRISMA flow diagram and the corresponding text have been carefully reviewed and corrected. The study selection process is now internally consistent, including the numbers of screened records, full-text articles assessed, excluded reports, and studies included in the final review.

Comment:
Attention should be paid to some repetition due to the new phrases added. Please see page 9, the final of the first and second paragraphs.

Response:
Thank you for this suggestion. The indicated paragraphs were revised to remove repetitive descriptions and improve readability while preserving the reported findings.

Comment:
Some details in references need to be replaced, namely the access date in Portuguese when the cited source is written in English.

Response:
We reviewed the reference list and corrected the formatting inconsistencies identified by the reviewer, including access-date language in references written in English.

**Response to Reviewer #2**

Comment:
The authors responded satisfactorily to the questions and clarifications provided. When posting to the system, I believe the cleaned manuscript was mistakenly attached, as it was the same manuscript from the initial submission.

Response:
We thank the reviewer for this observation. We believe that an incorrect clean version may have been uploaded during the previous revision process. For the current submission, we have carefully checked all files and uploaded both the revised manuscript with tracked changes and the updated clean manuscript containing all modifications described in this response.

**Additional Information**

In response to the journal requirements, we have also:

- Added an Author Summary to the manuscript.
- Updated and completed the CRediT author contribution statements in the submission system.
- Uploaded all figures as separate TIFF files.
- Added legends for all Supporting Information files.
- Provided supplementary tables containing the extracted study data, eligibility information, reasons for exclusion, and methodological quality assessment.
- Expanded the search strategy to include English, Portuguese, and Spanish descriptors.
- Updated the PRISMA flow diagram and corresponding text.
- Revised minor editorial issues throughout the manuscript.

We thank the Editor and Reviewers again for their valuable comments and consideration of our work.
