# Supplementary material for: Disability as a neglected outcome of neglected tropical diseases: A systematic review
Source: PLoS Negl Trop Dis. 2026 Jul 1;20(7):e0014480. doi: 10.1371/journal.pntd.0014480 (PMC13345467; doi:10.1371/journal.pntd.0014480)
Supplement: Peer review history

# Peer review: Decision Letter 0

**DOI:** 10.1371/journal.pntd.0014480.r001

**Authors:** Ran Wang, Max Eyre

19 Mar 2026

PNTD-D-26-00064

Disability as a neglected outcome of neglected tropical diseases: a systematic review

PLOS Neglected Tropical Diseases

Dear Dr. Goulart,

Thank you for submitting your manuscript to PLOS Neglected Tropical Diseases. After careful consideration, we feel that it has merit but does not fully meet PLOS Neglected Tropical Diseases's publication criteria as it currently stands. Therefore, we invite you to submit a revised version of the manuscript that addresses the points raised during the review process.

Please submit your revised manuscript within by May 18 2026 11:59PM. If you will need more time than this to complete your revisions, please reply to this message or contact the journal office at plosntds@plos.org. When you're ready to submit your revision, log on to https://www.editorialmanager.com/pntd/ and select the 'Submissions Needing Revision' folder to locate your manuscript file.

Please include the following items when submitting your revised manuscript:

* A letter that responds to each point raised by the editor and reviewer(s). You should upload this letter as a separate file labeled 'Response to Reviewers'. This file does not need to include responses to any formatting updates and technical items listed in the 'Journal Requirements' section below.

* A marked-up copy of your manuscript that highlights changes made to the original version. You should upload this as a separate file labeled 'Revised Manuscript with Track Changes'.

* An unmarked version of your revised paper without tracked changes. You should upload this as a separate file labeled 'Manuscript'.

If you would like to make changes to your financial disclosure, competing interests statement, or data availability statement, please make these updates within the submission form at the time of resubmission. Guidelines for resubmitting your figure files are available below the reviewer comments at the end of this letter.

We look forward to receiving your revised manuscript.

Kind regards,

Ran Wang, M.D.

Academic Editor

PLOS Neglected Tropical Diseases

Max Eyre

Section Editor

PLOS Neglected Tropical Diseases

Shaden Kamhawi

co-Editor-in-Chief

PLOS Neglected Tropical Diseases

orcid.org/0000-0003-4304-636XX

Paul Brindley

co-Editor-in-Chief

PLOS Neglected Tropical Diseases

orcid.org/0000-0003-1765-0002

**Additional Editor Comments :**

Overall, both reviewers consider the topic important and the manuscript potentially suitable for publication; however, substantial revisions are required to meet PLOS NTDs standards for systematic reviews.

The key priorities are to improve methodological transparency and reporting. Specifically, the authors should: (i) clearly define the research question (e.g., using PICO); (ii) clarify screening and data extraction procedures, including whether these were conducted in duplicate and how disagreements were resolved; (iii) ensure full compliance with PRISMA 2020, including protocol registration (or justification if not registered), complete and internally consistent flow diagram, and provision of full database-specific search strategies.

The authors must also address data availability requirements by depositing the extraction dataset and supporting materials (e.g., search strategies, study-level data, risk-of-bias assessments) in an open repository and updating the Data Availability statement with a DOI.

In addition, the manuscript would benefit from a more rigorous treatment of risk of bias and heterogeneity, including clearer justification of assessment tools, presentation of study-level quality assessments, and better integration of these into the interpretation (e.g., through stratified or sensitivity analyses).

Finally, the authors should resolve inconsistencies in reported numbers, improve clarity of tables/figures, and adopt a more cautious interpretation of headline estimates given the substantial conceptual heterogeneity and dominance of certain diseases and settings in the evidence base.

**Journal Requirements:**

1) Please ensure that the CRediT author contributions listed for every co-author are completed accurately and in full.At this stage, the following Authors/Authors require contributions: Caio Cesar Leiva Bastos Barrionuevo, Jefferson da Silva Valente, Bernardo Maia da Silva, Cássia da Luz Goulart, Alex Maciel, Aldair Darlan Santos-de-Araújo, Camila Miriam Suemi Sato Barros do Amaral, Eduardo Fernandes da Silva Junior, Stephanie Vitória Alves dos Santos, Erika Gomes, Nadia Cubas-Veja, Guilherme Peixoto Tinoco Arêas, and Fernando Almeida-Val. Please ensure that the full contributions of each author are acknowledged in the "Add/Edit/Remove Authors" section of our submission form.The list of CRediT author contributions may be found here: https://journals.plos.org/plosntds/s/authorship#loc-author-contributions 2) Please provide an Author Summary. This should appear in your manuscript between the Abstract (if applicable) and the Introduction, and should be 150-200 words long. The aim should be to make your findings accessible to a wide audience that includes both scientists and non-scientists. Sample summaries can be found on our website under Submission Guidelines:https://journals.plos.org/plosntds/s/submission-guidelines#loc-parts-of-a-submission 3) Please upload all main figures as separate Figure files in .tif or .eps format. For more information about how to convert and format your figure files please see our guidelines: https://journals.plos.org/plosntds/s/figures 4) We have noticed that you have uploaded Supporting Information files, but you have not included a list of legends. Please add a full list of legends for your Supporting Information files after the references list. 5) Some material included in your submission may be copyrighted. According to PLOS's copyright policy, authors who use figures or other material (e.g., graphics, clipart, maps) from another author or copyright holder must demonstrate or obtain permission to publish this material under the Creative Commons Attribution 4.0 International (CC BY 4.0) License used by PLOS journals. Please respond directly to this email and provide any known details concerning your material's license terms and permissions required for reuse, even if you have not yet obtained copyright permissions or are unsure of your material's copyright compatibility. Once you have responded and addressed all other outstanding technical requirements, you may resubmit your manuscript within Editorial Manager.  Potential Copyright Issues:i) Figure 3. Please confirm whether you drew the images / clip-art within the figure panels by hand. If you did not draw the images, please provide (a) a link to the source of the images or icons and their license / terms of use; or (b) written permission from the copyright holder to publish the images or icons under our CC BY 4.0 license. Alternatively, you may replace the images with open source alternatives. See these open source resources you may use to replace images / clip-art:- https://commons.wikimedia.org- https://openclipart.org/. ii) Figure 2. Please (a) provide a direct link to the base layer of the map (i.e., the country or region border shape) and ensure this is also included in the figure legend; and (b) provide a link to the terms of use / license information for the base layer image or shapefile. We cannot publish proprietary or copyrighted maps (e.g. Google Maps, Mapquest) and the terms of use for your map base layer must be compatible with our CC BY 4.0 license.Note: if you created the map in a software program like R or ArcGIS, please locate and indicate the source of the basemap shapefile onto which data has been plotted.If your map was obtained from a copyrighted source please amend the figure so that the base map used is from an openly available source. Alternatively, please provide explicit written permission from the copyright holder granting you the right to publish the material under our CC BY 4.0 license.If you are unsure whether you can use a map or not, please do reach out and we will be able to help you. The following websites are good examples of where you can source open access or public domain maps:* U.S. Geological Survey (USGS) - All maps are in the public domain. (http://www.usgs.gov)* PlaniGlobe - All maps are published under a Creative Commons license so please cite u201cPlaniGlobe, http://www.planiglobe.com, CC BY 2.0u201d in the image credit after the caption. (http://www.planiglobe.com/?lang=enl)* Natural Earth - All maps are public domain. (http://www.naturalearthdata.com/about/terms-of-use/).  6) We note that your Data Availability Statement is currently as follows: "No". Please provide a complete Data Availability Statement in the submission form, ensuring you include all necessary access information .

If your research concerns only data provided within your submission, please write "All data are in the manuscript and/or supporting information files" as your Data Availability Statement.

If your research concerns data from external sources, Please amend your Data Availability Statement to include the full link to the data. Please also confirm at this time whether or not your submission contains all raw data required to replicate the results of your study. Authors must share the “minimal data set” for their submission. PLOS defines the minimal data set to consist of the data required to replicate all study findings reported in the article, as well as related metadata and methods (https://journals.plos.org/plosone/s/data-availability#loc-minimal-data-set-definition).For example, authors should submit the following data: 1) The values behind the means, standard deviations and other measures reported;2) The values used to build graphs;3) The points extracted from images for analysis..Authors do not need to submit their entire data set if only a portion of the data was used in the reported study.If your submission does not contain these data, please either upload them as Supporting Information files or deposit them to a stable, public repository and provide us with the relevant URLs, DOIs, or accession numbers. For a list of recommended repositories, please see https://journals.plos.org/plosone/s/recommended-repositories. If there are ethical or legal restrictions on sharing a de-identified data set, please explain them in detail (e.g., data contain potentially sensitive information, data are owned by a third-party organization, etc.) and who has imposed them (e.g., an ethics committee). Please also provide contact information for a data access committee, ethics committee, or other institutional body to which data requests may be sent. If data are owned by a third party, please indicate how others may request data access. 7) As required by our policy on Data Availability, please ensure your manuscript or supplementary information includes the following:- A numbered table of all studies identified in the literature search, including those that were excluded from the analyses.- For every excluded study, the table should list the reason(s) for exclusion.- If any of the included studies are unpublished, include a link (URL) to the primary source or detailed information about how the content can be accessed.- A table of all data extracted from the primary research sources for the systematic review and/or meta-analysis. The table must include the following information for each study:  - Name of data extractors and date of data extraction  - Confirmation that the study was eligible to be included in the review.  - All data extracted from each study for the reported systematic review and/or meta-analysis that would be needed to replicate your analyses.  - If data or supporting information were obtained from another source (e.g. correspondence with the author of the original research article), please provide the source of data and dates on which the data/information were obtained by your research group.  - If applicable for your analysis, a table showing the completed risk of bias and quality/certainty assessments for each study or outcome.  Please ensure this is provided for each domain or parameter assessed. For example, if you used the Cochrane risk-of-bias tool for randomized trials, provide answers to each of the signalling questions for each study. If you used GRADE to assess certainty of evidence, provide judgements about each of the quality of evidence factor. This should be provided for each outcome.  - An explanation of how missing data were handled.This information can be included in the main text, supplementary information, or relevant data repository. Please note that providing these underlying data is a requirement for publication in this journal, and if these data are not provided your manuscript might be rejected.

Note: If the reviewer comments include a recommendation to cite specific previously published works, please review and evaluate these publications to determine whether they are relevant and should be cited. There is no requirement to cite these works unless the editor has indicated otherwise.

**Reviewers' Comments:**

Reviewer's Responses to Questions

**Key Review Criteria Required for Acceptance?**

As you describe the new analyses required for acceptance, please consider the following:

**Methods**

-Are the objectives of the study clearly articulated with a clear testable hypothesis stated?

-Is the study design appropriate to address the stated objectives?

-Is the population clearly described and appropriate for the hypothesis being tested?

-Is the sample size sufficient to ensure adequate power to address the hypothesis being tested?

-Were correct statistical analysis used to support conclusions?

-Are there concerns about ethical or regulatory requirements being met?

Reviewer #1: The hypothesis and the objectives are clear and appropriate to this systematic review. The study design is interesting, and several tools for systematic reviews are mentioned. The sample is large (130 studies found). Given the number of publications, this systematic review could deserve a meta-analysis. In addition, being a systematic review following PRISMA (2020) guidelines, some information is missing. Namely, the authors did not formulate the question of research with the PICO items. Moreover, the number of the protocol register in PROSPERO is not presented (Item 24 of PRISMA checklist), and it is a requirement for a true systematic review. In the search strategy and data sources, it is stated that no restrictions were applied for language and publication date, and information about supplementary materials is given. Thus, verifying this material, only English terms were used for the search, which suggests that only publications in the English language were obtained. So, here it seems to be a lack of coherence.

Regarding study selection, the number of studies in each step presented in the flow chart should be revised, since in the screening step, if 179 were excluded from the 480 records screened, it remains 301 (not 310). And excluded 180 from these, only 121 remain in the final.

Reviewer #2: Data Availability (PLOS policy)

The current Data Availability statement indicates that data are not available. For a systematic review, PLOS generally expects that the underlying extraction dataset and key materials are shared. Please deposit in an open repository (e.g., OSF/Zenodo/Figshare) at minimum:

a) the full extraction dataset (study-level variables and outcomes),

b) a data dictionary/codebook,

c) the final list of included studies, and preferably the list of excluded full texts with reasons,

d) risk-of-bias/quality assessment tables per study,

e) full search strategies per database and the date each search was run.

Please update the Data Availability statement accordingly and cite the repository accession/DOI in the manuscript.

Screening was performed by two reviewers with adjudication by a third reviewer; please clarify whether data extraction was also performed in duplicate (or how errors were minimized), and how disagreements were handled. Please consider reporting agreement for title/abstract screening and full-text eligibility assessment (e.g., Cohen’s kappa, weighted kappa, or percent agreement) and clarify whether data extraction was also conducted in duplicate. This would improve transparency and confidence in the review process and align with good reporting practices (e.g., PRISMA). If agreement metrics were not calculated, please provide a brief justification and describe any calibration/training steps and quality-control procedures used to minimize selection and extraction errors.

The manuscript uses MINORS for observational studies, CARE for case reports/series, and the Jadad scale for randomized controlled trials. This may be acceptable; however: MINORS is not currently the most commonly used instrument for observational studies in systematic reviews (many journals prefer ROBINS-I or the Newcastle–Ottawa Scale, depending on the study design). The Results section reports overall scores (e.g., “18–24/24”) but does not clearly show how risk of bias influenced the interpretation of the findings.

Suggestion: (i) provide a stronger justification for the choice of tools; (ii) present a study-by-study summary table stratified by design; and (iii) include a simple sensitivity analysis (e.g., excluding lower-quality studies or stratifying results by study design) to assess the impact on the main conclusions.

Was the review protocol registered in PROSPERO?

**********

**Results**

-Does the analysis presented match the analysis plan?

-Are the results clearly and completely presented?

-Are the figures (Tables, Images) of sufficient quality for clarity?

Reviewer #1: The type of analysis presented is appropriate to a systematic review, and tables and figures are of sufficient quality. However, some improvements are strongly recommended. In the flow chart (Figure 1), the number of duplicate records is 478, but at the beginning of the results, it is mentioned 345 duplicates. So, it’s important to verify all the numbers and replace where it is wrong.

Table 1, in the second column, presents “Author, Year”, but the studies have more than one author. The appropriate references should be indicated, for example, using “et al.” if more than three authors.

Reviewer #2: The review synthesizes a substantial body of literature (130 studies, 551,574 individuals, 25 countries), which supports the central argument that disability is a meaningful and under-recognized outcome across NTDs.

Presenting a descriptive estimate that 29% of assessed individuals had “at least one disability” is an effective headline for communicating relevance.

The Results highlight that physical/motor disability predominates (24.6%), followed by visual disability (1.81%), and they also mention cardiovascular, neurological, and psychosocial sequelae, which helps broaden the reader’s view beyond a single outcome type.

The manuscript explicitly notes heterogeneity and underreporting, which is essential framing for an evidence base spanning multiple diseases and instruments.

**********

**Conclusions**

-Are the conclusions supported by the data presented?

-Are the limitations of analysis clearly described?

-Do the authors discuss how these data can be helpful to advance our understanding of the topic under study?

-Is public health relevance addressed?

Reviewer #1: Given the results presented, the discussion and conclusions are in accordance with the same. Limitations are also presented, although they should be more in-depth. Here, it is evident that limitations are due essentially to the lack of a protocol to define the participant, interventions, comparisons and outcomes (PICO).

The study refers to the gap between disabilities associated to NTD, and the programs focused on NTDs. So, it is an important topic for public health.

In addition, some abbreviations need to be described before their use (for example, “NR” on page 11).

Reviewer #2: The review has several important limitations. First, there is substantial heterogeneity in how “disability” and “sequelae” are defined and measured across studies (different instruments, domains, and thresholds), which reduces comparability and limits the strength of quantitative synthesis. Second, the evidence base is dominated by leprosy and by a small number of countries/regions (notably Brazil), which may constrain generalizability to other NTDs and epidemiological settings. Many included studies are observational and facility-based, raising the risk of selection bias toward more severe cases or populations with better access to care, potentially inflating estimates of disability frequency and severity. Although risk-of-bias/quality assessments were conducted, their integration into the interpretation of key findings is limited. Finally, the lack of meta-analysis (likely justified by heterogeneity), underreporting and evidence gaps for several NTDs (especially psychosocial outcomes and longitudinal follow-up), as well as possible publication bias and under-coverage of grey literature, may have influenced the overall estimates and the profile of outcomes identified.

**********

**Editorial and Data Presentation Modifications?**

Use this section for editorial suggestions as well as relatively minor modifications of existing data that would enhance clarity. If the only modifications needed are minor and/or editorial, you may wish to recommend “Minor Revision” or “Accept”.

Reviewer #1: (No Response)

Reviewer #2: Minor Accept

**********

**Summary and General Comments**

Use this section to provide overall comments, discuss strengths/weaknesses of the study, novelty, significance, general execution and scholarship. You may also include additional comments for the author, including concerns about dual publication, research ethics, or publication ethics. If requesting major revision, please articulate the new experiments that are needed.

Reviewer #1: The text is well written and is easy to read and understand. In general, it is well organised for academic purposes. The topic is very interesting and reveals a needed research regarding the consequences of NTDs. The number of studies found is a strength of the study. However, some weaknesses were found given the type of study – systematic review. All the items of the PRISMA checklist must be fulfilled.

Reviewer #2: This systematic review addresses an important and under-recognized topic for PLOS NTDs: disability as a long-term outcome of NTDs. The search appears comprehensive (multiple databases, no language/date restrictions) and the manuscript follows PRISMA 2020, with a sizeable included evidence base. However, I recommend Major Revision primarily due to (i) Data Availability / Open Science compliance (the current Data Availability statement indicates “No”), which is inconsistent with PLOS policy for systematic reviews; (ii) insufficiently transparent reporting of full search strategies (per database), extraction workflow (double extraction/QA), and risk-of-bias integration; and (iii) conceptual heterogeneity in the operational definition of “disability/sequelae” that makes the headline prevalence summary (e.g., “29%”) potentially misleading without clearer stratification (ICF-based categories and/or measurement type). The narrative conclusions are valuable, but the manuscript needs stronger methodological transparency and more cautious interpretation given the dominance of leprosy/Brazilian studies in the included corpus. If these points are addressed, the study could make a strong contribution.

**********

PLOS authors have the option to publish the peer review history of their article (what does this mean?). If published, this will include your full peer review and any attached files.

If you choose “no”, your identity will remain anonymous but your review may still be made public.

**Do you want your identity to be public for this peer review?**  For information about this choice, including consent withdrawal, please see our Privacy Policy.

Reviewer #1: **Yes:**  Zélia Anastácio

Reviewer #2: No

[NOTE: If reviewer comments were submitted as an attachment file, they will be attached to this email and accessible via the submission site. Please log into your account, locate the manuscript record, and check for the action link "View Attachments". If this link does not appear, there are no attachment files.]

**Figure resubmission:**

While revising your submission, we strongly recommend that you use PLOS’s NAAS tool (https://ngplosjournals.pagemajik.ai/artanalysis) to test your figure files. NAAS can convert your figure files to the TIFF file type and meet basic requirements (such as print size, resolution), or provide you with a report on issues that do not meet our requirements and that NAAS cannot fix.

After uploading your figures to PLOS’s NAAS tool - https://ngplosjournals.pagemajik.ai/artanalysis, NAAS will process the files provided and display the results in the "Uploaded Files" section of the page as the processing is complete. If the uploaded figures meet our requirements (or NAAS is able to fix the files to meet our requirements), the figure will be marked as "fixed" above. If NAAS is unable to fix the files, a red "failed" label will appear above. When NAAS has confirmed that the figure files meet our requirements, please download the file via the download option, and include these NAAS processed figure files when submitting your revised manuscript.

**Reproducibility:**

To enhance the reproducibility of your results, we recommend that authors of applicable studies deposit laboratory protocols in protocols.io, where a protocol can be assigned its own identifier (DOI) such that it can be cited independently in the future. Additionally, PLOS ONE offers an option to publish peer-reviewed clinical study protocols. Read more information on sharing protocols at https://plos.org/protocols?utm_medium=editorial-email&utm_source=authorletters&utm_campaign=protocols

# Author comment: Author response to Decision Letter 1

**DOI:** 10.1371/journal.pntd.0014480.r002

28 Apr 2026

Attachment Submitted filename: Response to Reviewers Caio-2.docx

# Peer review: Decision Letter 1

**DOI:** 10.1371/journal.pntd.0014480.r003

**Authors:** Ran Wang, Max Eyre, Ran Wang, Max Eyre

28 May 2026

PNTD-D-26-00064R1

Disability as a neglected outcome of neglected tropical diseases: a systematic review

PLOS Neglected Tropical Diseases

Dear Dr. Goulart,

Thank you for submitting your manuscript to PLOS Neglected Tropical Diseases. After careful consideration, we feel that it has merit but does not fully meet PLOS Neglected Tropical Diseases's publication criteria as it currently stands. Therefore, we invite you to submit a revised version of the manuscript that addresses the points raised during the review process.

Please submit your revised manuscript by Jun 27 2026 11:59PM. If you will need more time than this to complete your revisions, please reply to this message or contact the journal office at plosntds@plos.org. When you're ready to submit your revision, log on to https://www.editorialmanager.com/pntd/ and select the 'Submissions Needing Revision' folder to locate your manuscript file.

Please include the following items when submitting your revised manuscript:

* A letter that responds to each point raised by the editor and reviewer(s). You should upload this letter as a separate file labeled 'Response to Reviewers'. This file does not need to include responses to any formatting updates and technical items listed in the 'Journal Requirements' section below.

* A marked-up copy of your manuscript that highlights changes made to the original version. You should upload this as a separate file labeled 'Revised Manuscript with Track Changes'.

* An unmarked version of your revised paper without tracked changes. You should upload this as a separate file labeled 'Manuscript'.

If you would like to make changes to your financial disclosure, competing interests statement, or data availability statement, please make these updates within the submission form at the time of resubmission. Guidelines for resubmitting your figure files are available below the reviewer comments at the end of this letter.

As the corresponding author, your ORCID iD is verified in the submission system and will appear in the published article. PLOS supports the use of ORCID, and we encourage all coauthors to register for an ORCID iD and use it as well. Please encourage your coauthors to verify their ORCID iD within the submission system before final acceptance, as unverified ORCID iDs will not appear in the published article. Only the individual author can complete the verification step; PLOS staff cannot verify ORCID iDs on behalf of authors.

We look forward to receiving your revised manuscript.

Kind regards,

Ran Wang, M.D.

Academic Editor

PLOS Neglected Tropical Diseases

Max Eyre

Section Editor

PLOS Neglected Tropical Diseases

Shaden Kamhawi

co-Editor-in-Chief

PLOS Neglected Tropical Diseases

orcid.org/0000-0003-4304-636XX

Paul Brindley

co-Editor-in-Chief

PLOS Neglected Tropical Diseases

orcid.org/0000-0003-1765-0002

**Additional Editor Comments:**

Please could you clarify regarding the languages in which search terms were searched for, in response to this reviewer comment: 'Given the higher number of Brazilian studies and the author's declaration that "No restrictions were applied regarding language or publication date", it is strange that there are no other terms than the English MeSH, instead of 47 of the 161 references being in Portuguese or Spanish language. Probably, the authors have a justification for it, but it is not clear in the text."

**Journal Requirements:**

If the reviewer comments include a recommendation to cite specific previously published works, please review and evaluate these publications to determine whether they are relevant and should be cited. There is no requirement to cite these works unless the editor has indicated otherwise.

1) Please ensure that the CRediT author contributions listed for every co-author are completed accurately and in full.

At this stage, the following Authors/Authors require contributions: Caio Cesar Leiva Bastos Barrionuevo, Jefferson da Silva Valente, Bernardo Maia da Silva, Cássia da Luz Goulart, Alex Maciel, Aldair Darlan Santos-de-Araújo, Camila Miriam Suemi Sato Barros do Amaral, Eduardo Fernandes da Silva Junior, Stephanie Vitória Alves dos Santos, Erika Gomes, Nadia Cubas-Veja, Guilherme Peixoto Tinoco Arêas, and Fernando Almeida-Val. Please ensure that the full contributions of each author are acknowledged in the "Add/Edit/Remove Authors" section of our submission form.

The list of CRediT author contributions may be found here: https://journals.plos.org/plosntds/s/authorship#loc-author-contributions

2) Your Response to Reviewers should be uploaded as a single, individual file. Please remove your Response to Reviewers from your Cover Letter.

3) Please provide an Author Summary. This should appear in your manuscript between the Abstract (if applicable) and the Introduction, and should be 150-200 words long. The aim should be to make your findings accessible to a wide audience that includes both scientists and non-scientists. Sample summaries can be found on our website under Submission Guidelines:

https://journals.plos.org/plosntds/s/submission-guidelines#loc-parts-of-a-submission

4) Please upload all main figures as separate Figure files in .tif or .eps format. For more information about how to convert and format your figure files please see our guidelines:

https://journals.plos.org/plosntds/s/figures

5) We have noticed that you have uploaded Supporting Information files, but you have not included a list of legends. Please add a full list of legends for your Supporting Information files after the references list.

6) As required by our policy on Data Availability, please ensure your manuscript or supplementary information includes the following:

- A numbered table of all studies identified in the literature search, including those that were excluded from the analyses.

- For every excluded study, the table should list the reason(s) for exclusion.

- If any of the included studies are unpublished, include a link (URL) to the primary source or detailed information about how the content can be accessed.

- A table of all data extracted from the primary research sources for the systematic review and/or meta-analysis. The table must include the following information for each study:

- Name of data extractors and date of data extraction

- Confirmation that the study was eligible to be included in the review.

- All data extracted from each study for the reported systematic review and/or meta-analysis that would be needed to replicate your analyses.

- If data or supporting information were obtained from another source (e.g. correspondence with the author of the original research article), please provide the source of data and dates on which the data/information were obtained by your research group.

- If applicable for your analysis, a table showing the completed risk of bias and quality/certainty assessments for each study or outcome.  Please ensure this is provided for each domain or parameter assessed. For example, if you used the Cochrane risk-of-bias tool for randomized trials, provide answers to each of the signalling questions for each study. If you used GRADE to assess certainty of evidence, provide judgements about each of the quality of evidence factor. This should be provided for each outcome.

- An explanation of how missing data were handled.

This information can be included in the main text, supplementary information, or relevant data repository. Please note that providing these underlying data is a requirement for publication in this journal, and if these data are not provided your manuscript might be rejected.

**Reviewers' comments:**

Reviewer's Responses to Questions

**Key Review Criteria Required for Acceptance?**

As you describe the new analyses required for acceptance, please consider the following:

**Methods**

-Are the objectives of the study clearly articulated with a clear testable hypothesis stated?

-Is the study design appropriate to address the stated objectives?

-Is the population clearly described and appropriate for the hypothesis being tested?

-Is the sample size sufficient to ensure adequate power to address the hypothesis being tested?

-Were correct statistical analysis used to support conclusions?

-Are there concerns about ethical or regulatory requirements being met?

Reviewer #1: Methods were partially improved: authors referred to the PROSPERO number registration (instead of not having the direct link to it). A paragraph explaining the research question was introduced. However, the research question itself is still missing. Given the higher number of Brazilian studies and the author's declaration that "No restrictions were applied regarding language or publication date", it is strange that there are no other terms than the English MeSH, instead of 47 of the 161 references being in Portuguese or Spanish language. Probably, the authors have a justification for it, but it is not clear in the text.

As I referred in the first review, "Regarding study selection, the number of studies in each step presented in the flow chart should be revised, since in the screening step, if 179 were excluded from the 480 records screened, it remains 301 (not 310). And excluded 180 from these, only 121 remain in the final." In the answer, the authors referred to the replacement in the PRSIMA Flow Chart, but it remains equal.

Reviewer #2: The authors responded satisfactorily to the questions and clarifications provided. When posting to the system, I believe the cleaned manuscript was mistakenly attached, as it was the same manuscript from the initial submission, which does not have the adjustments indicated in the file with the changes highlighted (Revised Article with Changes Highlighted).

**********

**Results**

-Does the analysis presented match the analysis plan?

-Are the results clearly and completely presented?

-Are the figures (Tables, Images) of sufficient quality for clarity?

Reviewer #1: The results are good. The authors improved it by replacing "Author, Year" in the table by "First author, Year", as they justified in their answer. Attention should be paid to some repetition due to the new phrases added. Please, see page 9, the final of the 1st and 2nd paragraphs.

Reviewer #2: The authors responded satisfactorily to the questions and clarifications provided. When posting to the system, I believe the cleaned manuscript was mistakenly attached, as it was the same manuscript from the initial submission, which does not have the adjustments indicated in the file with the changes highlighted (Revised Article with Changes Highlighted).

**********

**Conclusions**

-Are the conclusions supported by the data presented?

-Are the limitations of analysis clearly described?

-Do the authors discuss how these data can be helpful to advance our understanding of the topic under study?

-Is public health relevance addressed?

Reviewer #1: Conclusions are supported by the results obtained, limitations are presented, and my question about the meta-analysis is answered in a good sense. Discussion mobilises other literature for the present study and is in accordance with the results.

Reviewer #2: The authors responded satisfactorily to the questions and clarifications provided. When posting to the system, I believe the cleaned manuscript was mistakenly attached, as it was the same manuscript from the initial submission, which does not have the adjustments indicated in the file with the changes highlighted (Revised Article with Changes Highlighted).

**********

**Editorial and Data Presentation Modifications?**

Use this section for editorial suggestions as well as relatively minor modifications of existing data that would enhance clarity. If the only modifications needed are minor and/or editorial, you may wish to recommend “Minor Revision” or “Accept”.

Reviewer #1: In my opinion, the text needs minor revision as detailed above and in the attached manuscript.

Reviewer #2: Accept

**********

**Summary and General Comments**

Use this section to provide overall comments, discuss strengths/weaknesses of the study, novelty, significance, general execution and scholarship. You may also include additional comments for the author, including concerns about dual publication, research ethics, or publication ethics. If requesting major revision, please articulate the new experiments that are needed.

Reviewer #1: The study is interesting, approaches an important issue and raises an alert for sub-notified NTDs. Several improvements were made, as well as authors provide additional information to justify their choices. Some minor improvements are recommended for a better quality of the manuscript. Some little details in references also need to be replaced, namely the access date in Portuguese, when it is a text in the English language.

Reviewer #2: The authors responded satisfactorily to the questions and clarifications provided. When posting to the system, I believe the cleaned manuscript was mistakenly attached, as it was the same manuscript from the initial submission, which does not have the adjustments indicated in the file with the changes highlighted (Revised Article with Changes Highlighted).

**********

PLOS authors have the option to publish the peer review history of their article (what does this mean?). If published, this will include your full peer review and any attached files.

If you choose “no”, your identity will remain anonymous but your review may still be made public.

**Do you want your identity to be public for this peer review?**  For information about this choice, including consent withdrawal, please see our Privacy Policy.

Reviewer #1: **Yes:** Zélia Anastácio

Reviewer #2: No

[NOTE: If reviewer comments were submitted as an attachment file, they will be attached to this email and accessible via the submission site. Please log into your account, locate the manuscript record, and check for the action link "View Attachments". If this link does not appear, there are no attachment files.]

**Figure resubmission:**

While revising your submission, we strongly recommend that you use PLOS’s NAAS tool (https://ngplosjournals.pagemajik.ai/artanalysis) to test your figure files. NAAS can convert your figure files to the TIFF file type and meet basic requirements (such as print size, resolution), or provide you with a report on issues that do not meet our requirements and that NAAS cannot fix.

After uploading your figures to PLOS’s NAAS tool - https://ngplosjournals.pagemajik.ai/artanalysis, NAAS will process the files provided and display the results in the "Uploaded Files" section of the page as the processing is complete. If the uploaded figures meet our requirements (or NAAS is able to fix the files to meet our requirements), the figure will be marked as "fixed" above. If NAAS is unable to fix the files, a red "failed" label will appear above. When NAAS has confirmed that the figure files meet our requirements, please download the file via the download option, and include these NAAS processed figure files when submitting your revised manuscript.

**Reproducibility:**

To enhance the reproducibility of your results, we recommend that authors of applicable studies deposit laboratory protocols in protocols.io, where a protocol can be assigned its own identifier (DOI) such that it can be cited independently in the future. Additionally, PLOS ONE offers an option to publish peer-reviewed clinical study protocols. Read more information on sharing protocols at https://plos.org/protocols?utm_medium=editorial-email&utm_source=authorletters&utm_campaign=protocols

Attachment Submitted filename: Manuscrito_Caio_01-04-2026_Rev2.docx

# Author comment: Author response to Decision Letter 2

**DOI:** 10.1371/journal.pntd.0014480.r004

11 Jun 2026

Attachment Submitted filename: (update) response to the reviewers.docx

# Editor report: Decision Letter 2

**DOI:** 10.1371/journal.pntd.0014480.r005

**Authors:** Ran Wang, Max Eyre, Ran Wang, Max Eyre, Ran Wang, Max Eyre

18 Jun 2026

Dear Dr Almeida-Val,

We are pleased to inform you that your manuscript 'Disability as a neglected outcome of neglected tropical diseases: a systematic review' has been provisionally accepted for publication in PLOS Neglected Tropical Diseases.

Before your manuscript can be formally accepted you will need to complete some formatting changes, which you will receive in a follow up email. A member of our team will be in touch with a set of requests.

Please note that your manuscript will not be scheduled for publication until you have made the required changes, so a swift response is appreciated.

IMPORTANT: The editorial review process is now complete. PLOS will only permit corrections to spelling, formatting or significant scientific errors from this point onwards. Requests for major changes, or any which affect the scientific understanding of your work, will cause delays to the publication date of your manuscript.

Should you, your institution's press office or the journal office choose to press release your paper, you will automatically be opted out of early publication. We ask that you notify us now if you or your institution is planning to press release the article. All press must be co-ordinated with PLOS.

Thank you again for supporting Open Access publishing; we are looking forward to publishing your work in PLOS Neglected Tropical Diseases.

Best regards,

Max Thomas Eyre, PhD

Section Editor

PLOS Neglected Tropical Diseases

Max Eyre

Section Editor

PLOS Neglected Tropical Diseases

Shaden Kamhawi

co-Editor-in-Chief

PLOS Neglected Tropical Diseases

orcid.org/0000-0003-4304-636XX

Paul Brindley

co-Editor-in-Chief

PLOS Neglected Tropical Diseases

orcid.org/0000-0003-1765-0002

***********************************************************

# Editor report: Acceptance letter

**DOI:** 10.1371/journal.pntd.0014480.r006

**Authors:** Ran Wang, Max Eyre, Ran Wang, Max Eyre, Ran Wang, Max Eyre

Dear Dr Almeida-Val,

We are delighted to inform you that your manuscript, "Disability as a neglected outcome of neglected tropical diseases: a systematic review," has been formally accepted for publication in PLOS Neglected Tropical Diseases.

We have now passed your article onto the PLOS Production Department who will complete the rest of the publication process. All authors will receive a confirmation email upon publication.

The corresponding author will soon be receiving a typeset proof for review, to ensure errors have not been introduced during production. Please review the PDF proof of your manuscript carefully, as this is the last chance to correct any scientific or type-setting errors. Please note that major changes, or those which affect the scientific understanding of the work, will likely cause delays to the publication date of your manuscript. Note: Proofs for Front Matter articles (Editorial, Viewpoint, Symposium, Review, etc...) are generated on a different schedule and may not be made available as quickly.

Soon after your final files are uploaded, the early version of your manuscript will be published online unless you opted out of this process. The date of the early version will be your article's publication date. The final article will be published to the same URL, and all versions of the paper will be accessible to readers.

For Research Articles, you will receive an invoice from PLOS for your publication fee after your manuscript has reached the completed accept phase. If you receive an email requesting payment before acceptance or for any other service, this may be a phishing scheme. Learn how to identify phishing emails and protect your accounts at https://explore.plos.org/phishing.

Thank you again for supporting open-access publishing; we are looking forward to publishing your work in PLOS Neglected Tropical Diseases.

Best regards,

Shaden Kamhawi

co-Editor-in-Chief

PLOS Neglected Tropical Diseases

Paul Brindley

co-Editor-in-Chief

PLOS Neglected Tropical Diseases
